# Optical imaging probes in oncology

**DOI:** 10.18632/oncotarget.9066

**Published:** 2016-04-27

**Authors:** Cristina Martelli, Alessia Lo Dico, Cecilia Diceglie, Giovanni Lucignani, Luisa Ottobrini

**Affiliations:** ^1^ Department of Pathophysiology and Transplantation, University of Milan, Milan, Italy; ^2^ Centre of Molecular and Cellular Imaging-IMAGO, Milan, Italy; ^3^ Umberto Veronesi Foundation, Milan, Italy; ^4^ Tecnomed Foundation, University of Milan-Bicocca, Monza, Italy; ^5^ Department of Health Sciences, University of Milan, Milan, Italy; ^6^ Institute for Molecular Bioimaging and Physiology (IBFM), National Research Council (CNR), Milan, Italy

**Keywords:** cancer, fluorescent probes, biomarkers, molecular processes, tumor cell features

## Abstract

Cancer is a complex disease, characterized by alteration of different physiological molecular processes and cellular features. Keeping this in mind, the possibility of early identification and detection of specific tumor biomarkers by non-invasive approaches could improve early diagnosis and patient management.

Different molecular imaging procedures provide powerful tools for detection and non-invasive characterization of oncological lesions. Clinical studies are mainly based on the use of computed tomography, nuclear-based imaging techniques and magnetic resonance imaging. Preclinical imaging in small animal models entails the use of dedicated instruments, and beyond the already cited imaging techniques, it includes also optical imaging studies. Optical imaging strategies are based on the use of luminescent or fluorescent reporter genes or injectable fluorescent or luminescent probes that provide the possibility to study tumor features even by means of fluorescence and luminescence imaging. Currently, most of these probes are used only in animal models, but the possibility of applying some of them also in the clinics is under evaluation.

The importance of tumor imaging, the ease of use of optical imaging instruments, the commercial availability of a wide range of probes as well as the continuous description of newly developed probes, demonstrate the significance of these applications. The aim of this review is providing a complete description of the possible optical imaging procedures available for the non-invasive assessment of tumor features in oncological murine models. In particular, the characteristics of both commercially available and newly developed probes will be outlined and discussed.

## INTRODUCTION

Cancer is a heterogeneous disease and different causal factors and processes are involved in its growth and progression. The possibility of early detection and characterization of tumor is of the utmost importance, because it increases the chances of treatment and cure.

Imaging techniques, permitting to obtain non-invasive images from the body, may help in the identification and localization of tumor lesions and in the characterization of processes involved in tumor proliferation and invasion, concurring to the description of tumor features, for a correct staging and the identification of the most efficacious treatment procedure and schedule.

Specific molecules, capable of tracing a precise event or a well-defined marker, have been labeled with different kinds of contrast agents, such as radionuclides for nuclear based imaging, paramagnetic or electron opaque substances for radiological techniques, or bioluminescent or fluorescent molecules for optical imaging. The signal generated by contrast agents are then detected with the dedicated imaging technique.

In the clinics, nuclear-based and radiological techniques are the most used procedures to identify tumor presence, and to monitor growth, staging and response to therapy. Even if new injectable fluorescent probes have been proposed for clinical use, their application is still limited. In pre-clinical oncological studies, in addition to magnetic resonance imaging, ultrasound and nuclear based imaging, optical imaging procedures can be affordable, using non-ionizing radiation and allowing a high-throughput analysis of small animal models expressing specific reporters (Luciferase or red and near-infrared fluorescent proteins) or injected with specific fluorescent (or chemiluminescent) probes. Moreover, optical imaging instrumentation have been proposed for a novel application based on the acquisition of Čerenkov [1] radiation emitted by charged particles, such as radionuclides/radiotracers.

**Figure 1:**
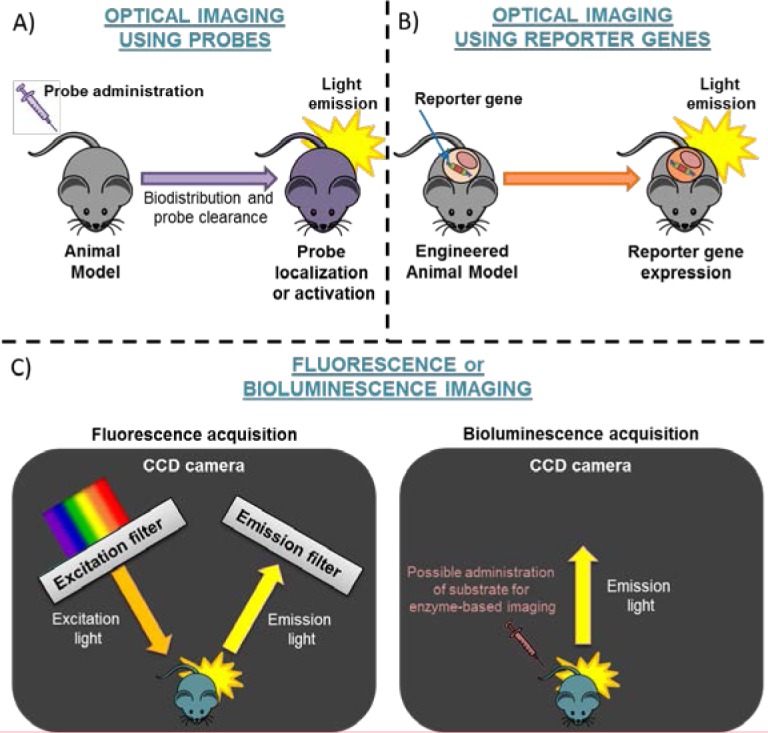
Schematic representation of Optical Imaging The strategies for optical imaging are based on: **A.** administration of probes or **B.** use of engineered mice expressing a reporter gene. Panel **C.** shows Fluorescence (dark box on the left side) or Bioluminescence Acquisition (dark box on the right side). CCD camera=Charged-Coupled Device camera.

**Figure 2:**
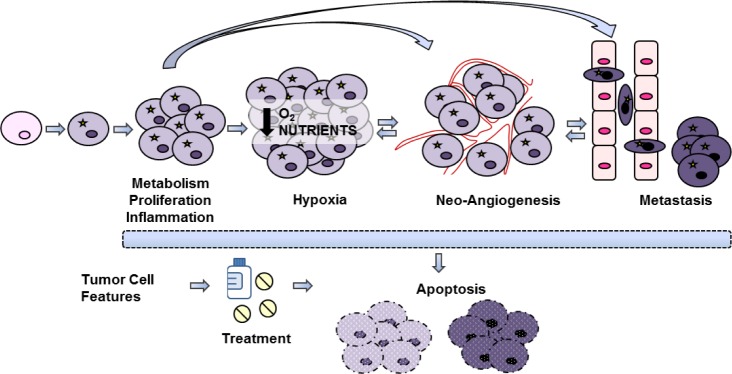
Tumor processes Schematic representation of different processes involved in tumor growth and progression, that can be visualized by using imaging techniques.

Optical imaging procedures include, in fact, different imaging possibilities such as: fluorescence (FLI), bioluminescence (BLI) (Figure 1) and Čerenkov (CLI) imaging. In this review, first of all the different strategies will be described and discussed, then the possibility of visualizing different tumor cell processes and cellular features using specific optical probes will be highlighted (Figure 2). Finally, a further section will explain the possibility of using these strategies for newly discovered cellular targets.

### Fluorescence imaging (FLI)

Fluorescent imaging [2–4] is based on the detection of photons (wavelength range: 442-800 nm) produced by the return to ground state of electrons excited by photostimulation. These photons can be revealed by a cooled CCD-camera by using specific filters to select excitation and emission band and to reduce noise background.

Fluorescence can be obtained through the use of fluorophores or reporter genes. The first strategy, in the same way of nuclear based imaging, in which a probe is labeled with a radiotracer, entails use of a fluorescent molecule able to report the localization of the conjugated probe. The second strategy is based instead on the use of reporter genes (such as Red Fluorescent Proteins -RFP, or mCherry) able to provide information about a specific process or molecular event (by using a specific promoter or by direct linking with a key molecule). Even if the first strategy is influenced by the size of fluorophore, that could affect the targeting or the biodistribution of the probes, it is easier to use compared to the second, because it does not require cell engineering to insert the reporter gene into the genome (the reporter molecules could also be conjugated to the probe, but there would be a significant increase in size).

FLI has several advantages: first, it does not require the administration of a substrate that has to be enzymatically modified to emit photons. Second, it allows to exploit different fluorophores with different spectra of excitation and emission (Table 1), characterized by high quantum yield and high penetration in tissues, to acquire multiple signals monitoring different processes in the same experimental animal. Third, the same fluorescent signal can be used for the *in vivo* monitoring of a specific molecular process and its *ex vivo* validation by fluorescence microscopy. Last but not least, the availability of several specific probes for different processes has eliminated the need of developing new reporter gene expressing models, speeding up the translation of pre-clinical data into the clinics.

**Table 1:** List of fluotochromes used to label molecules (in order of emission wavelength)

FLUOROCHROME	λ_max_ EXCITATION (nm)	λ_max_ EMISSION (nm)
Bodipy-FL	480	513
5-Carboxyfluorescein	492	517
AF488	498	520
RhodamineG	525	555
QD580	350	580
QD590	350	590
NANQ	463	615
QD645	350	645
645	649	666
Pyro a	412/670	675
AF680	675	693
Cy5.5	675	695
DY-676	676	705
QD705	350	705
DY-680	677	705
Cy7	743	767
ITCC	740	770
DY-750	751	772
AF750	755	775
IRD800	775	792
QD800	350	800
ICG	785	801
X-SIGHT760	760	813
QD820	350	820
QD840	350	840

Among disadvantages, FLI requires the use of an excitation light, that could be attenuated by the tissues with a consequent lower excitation efficiency of deeply located fluorochromes, and it is characterized by a significant amount of auto-fluorescence which determines a high background level due to the presence of endogenously fluorescent molecules. For this reason, the quantification of FL signal can be often difficult.

### Bioluminescence imaging (BLI)

Bioluminescence [2,5] or chemiluminescence [6] imaging are based on the detection of photons produced by a chemical reaction: A + B → C + light, where A and B could be an enzyme and its substrate and C is the product(s) of the reaction. In this case the cooled CCD-camera does not need filters, revealing all the photons emitted by the sample.

BLI is mainly based on the use of reporter genes encoding enzymes, as a rule Luciferase, able to emit photons in the presence of specific substrates, such as D-Luciferin for Firefly Luciferase or coelenterazine for Renilla Luciferase, in the wavelength range of 485-613 nm, or reporter probes labeled with these enzymes or conjugated with chemi-luminescent molecules, such as luminol.

Among the major advantages of BLI, there is the independence from an excitation light (reducing the photon attenuation, scattering and diffusion when compared with FLI, since photons cross tissues only once), and the absence of background signal due to luminescence emitting endogenous molecules, determining a more accurate signal quantification, resulting to be dependent by the number of luciferase expressing cells or the enzymatic chemiluminescent reaction.

BLI disadvantages are the requirement of substrate administration, the low penetration of emitted photons, and sometimes the longer acquisition time compared to fluorescence imaging (ranging from 1 second to 5 minutes for BLI imaging and from 1 second to 60 seconds for FLI imaging). Moreover, despite the large use of Luciferase as reporter gene for *in vitro* and *in vivo* imaging procedures, the requirement of cell engineering and the large size of bioluminescent enzymes for probe conjugation makes this strategy less appealing for the easy and fast visualization of oncological processes and features, even if some examples are given in this review.

### Čerenkov luminescence imaging (CLI)

The Čerenkov Imaging is based on the detection of photons emitted by a charged particle that moves through a dielectric medium at a speed higher than the velocity of light in that medium [1]. In molecular imaging applications, this process can be generated by high speed Compton electrons produced by gamma radiation (derived by nuclear decay of isotopes already used for nuclear based imaging techniques such as ^18^F, ^11^C, ^99m^Tc and ^131^I), and an ultra-sensitive CCD camera for BLI can be used to reveal this visible light.

Čerenkov radiation can be used for non-invasive monitoring by CCD camera imaging of a radionuclide labeled tracer localization, or, instead of directly analyze Čerenkov radiation itself, the emission can be used as an internal excitation source for other fluorophores. The latter strategy permits the detection of signals with higher penetration compared to the former one, but it is less used.

There are several potential applications in the clinics and advantages of this kind of Optical Imaging procedure. The possibility to quantify in real time α and beta decay allows measuring dosimetry for a specific target organ during radiotherapy. The same radiation could be used during surgery or during endoscopy to drive tumor resection, in small animal tomography, or for the monitoring of pH or O_2_ levels [7–8].

The short range of photon penetration in tissues restricts whole body CLI to small animal imaging; human use is limited to near surface emissions such as visualization of thyroid [9], that showed a good agreement with PET scan.

Despite the recent importance acquired by this technique, examples of Čerenkov Imaging will not be provided in this review, which focuses on the use of non-radioactive probes.

### Molecular sensitivity

The molecular sensitivity of an imaging technique is defined, from CS Levin, as the “capability to detect, visualize and accurately quantify low concentration of molecular probe interacting with a molecular target on or within cells of a living subject” [10]. In other words, sensitivity is the lower signal that can be detected by a specific imaging modality.

Considering optical imaging, FLI is characterized by a typical molecular sensitivity value that is over an order of magnitude lower compared to BLI (10^−9^ ÷ 10^−11^ and 10^−13^ ÷ 10^−16^, respectively [11]).

Molecular sensitivity has been described by Levin to be dependent “by a combination of the probe and biological/physiological properties of the subject that determine its specificity for the target and the performance capabilities of the imaging system that determine how well the resulting signal can be detected and measured” [10]. Considering the latter part, some physical parameters should be taken into account to precisely define molecular sensitivity. It strongly depends on quantum efficiency and geometry of light sensor, probability of photons conversion to electric charge, background dark current (mainly due to sensor temperature), and the setting of acquisition parameters (binning, pixel size, exposure time, position of field of view). Most of these parameters influence sensitivity both for FLI and BLI, e.g. excitation light used in FLI is also involved by influencing quantum efficiency of light sensors, and can vary greatly from one imaging system to another.

In addition to the specificity of probe-target interaction, that will be explained in the following paragraphs, biological background signal is the other significant variable to consider if we analyze molecular sensitivity. Background signal in FLI acquisitions is particularly important since it can be due to both auto-fluorescence of endogenous fluorescent molecules present in the cells and tissues and by the a-specific uptake of a fluorophore labeled probe and can significantly vary specific fluorescence acquisition.

## MOLECULAR PROCESSES

### Metabolism

Metabolism is defined as the sum of the physical and chemical processes in a cell able to produce, maintain and destroy materials, and by which energy is made available. All metabolic processes are finely regulated into the cells, and studies carried out on tumor samples and clinical trials have indicated a strong relationship between activation of oncogenes and modification in cell metabolism. Cell metabolism modification is, in fact, a common tumor feature involving glucose, amino acid and/or lipid metabolism deregulation and can be considered as a surrogate biomarker correlated with tumor grade, proliferation rate, aggressiveness, etc. Molecular imaging highlighted the importance to visualize tumor metabolism, because this information could influence patient management, by improving tumor staging, re-staging, radiation treatment planning, and monitoring of tumor response to therapy [12–13].

Regarding glucose metabolism, glycolysis is inhibited, in normal mammalian cells, by the presence of oxygen, which allows mitochondria to oxidize pyruvate to CO_2_ and H_2_O (Pasteur Effect); however, in cancer cells glycolysis is increased, and glucose is converted into lactic acid in presence of oxygen (a process that is called aerobic glycolysis). As firstly reported by Otto Warburg at the beginning of the 20^th^ century, this is a specific metabolic abnormality of cancer cells.

Glucose was firstly labeled with ^14^C and following with ^18^F to study glucose metabolism by PET, and to date, ^18^F-FDG is the most used radiotracer for PET imaging. In order to study glucose uptake by fluorescence imaging, a more cost-effective, convenient, and high-throughput alternative to FDG-PET in pre-clinical imaging, glucose was also labeled with fluorophores [14]. The fluorescent 2-deoxy-D-glucose (2-DG, commercially available by PerkinElmer Life Sciences, Inc, Boston, MA, USA and by LI-COR Biotechnology - GmbH, Bad Homburg, Germany) has been generated by substitution of 2-hydroxyl group with various fluorophores emitting in the near infra-red (NIR) window (Cy5.5, ICG, IRD, etc.). Different studies [15–18] have reported the possibility to use 2-DG conjugates to efficiently visualize tumor masses in oncological murine models, with high selectivity of localization and retention in the lesions, even if the conjugation of 2-DG with NIR-fluorophores determines a significant increase of molecular weight compared to glucose, due to the high dimension of the fluorophore. In the works reported above, *in vivo* accumulation of this tracer seemed to reflect the presence of GLUT-1 (as for FDG uptake) on cell membrane, even if this transporter is not the only factor that can influence probe uptake. In contrast, in 2012, another work [19] reported the incongruity between [^18^F]FDG-PET and NIR-2DG imaging in a preclinical model of gastrointestinal stromal tumor (GIST). GIST bearing mice were treated with nilotinib, a c-kit inhibitor used in clinical practice that blocks glucose metabolism, and were monitored for ^18^F-FDG and NIR-2DG uptake. Images proved that fluorescence imaging with NIR-2DG probe did not change after treatment compared to the control groups. Conversely, ^18^F-FDG uptake, as expected, was found significantly reduced after nilotinib treatment showing the lack of correlation in the images provided by the two tracers. These findings indicated that NIR-2DG was able to detect tumors, but was not correlated to metabolic status. Despite this limitation, NIR-2DG fluorescent probe may be useful for assessing tumor bulk, even if it cannot substitute ^18^F-FDG PET in the study of tumor metabolism in response to treatment. For *in vivo* evaluation of lipid and amino acid metabolism, alternatives to nuclear imaging are not currently available.

### Proliferation

Proliferation is a tightly regulated process, which involves several proteins able to activate or block the cell cycle progression. Most tumor cells lose this fine regulation resulting in an abnormal neoplastic tissue growth [20]. Proliferation rate has been proposed as a biomarker for tumor grade, aggressiveness and tumor response to treatments [21–22]. Ki67 is the main biomarker used to *ex vivo* assess proliferation rate in immune histochemical analyses.

In the clinical setting, PET imaging with [^18^F-FLT] (3′-deoxy-3′-fluorothymidine) [23] is used to non-invasively monitor tumor cell proliferation, for example in the assessment of treatment. This tracer reports on Thymidine kinase activity in the salvage nucleotide biosynthesis pathway, providing an indirect evaluation of proliferation rate. Despite their clinical use and advantages, thymidine analogs have some pitfalls, such as the asynchronous cell cycle of tumor cells, the a-specific uptake by bone marrow, and the *de novo* thymidine pathway utilization, that might underestimate the real proliferation index [24–25].

To facilitate the study of tumor proliferation in pre-clinical models, overcoming the problems associated with the use of thymidine analogues, two novel fluorescent probes have been developed. The first one, the BombesinRsense680, generated by PerkinElmer, comprises a 7-amino acid bombesin peptide analog, a NIR fluorophore and a molecule to stabilize its plasma availability. This probe is designed to bind to the Bombesin specific receptor which is involved in stimulation of cancer cell proliferation. In fact, bombesin peptides act as autocrine growth factors, inducing either calcium mobilization or proliferation in tumor cells. This probe showed a significant accumulation in subcutaneous xenografts of colorectal HT-29 implanted in nude mice, with pancreas, skin and glands as secondary sites of uptake, due to endogenous expression of Bombesin receptor. It is characterized by short half-life in blood due to rapid clearance *via* kidneys and bladder [26]. The second commercially available proliferation probe for fluorescence imaging is called Tetra and was developed by Carestream Molecular Imaging (Carestream Health, Inc., Rochester, NY, USA) [27]. As showed by the company, the probe localizes in the tumor mass as demonstrated by its co-localization with X-SIGHT760 dye-labeled tumor cells. However, up to now, no elucidation about mechanism of action or its target has been provided and no *in vivo* studies using this probe have been published.

Due to the continuous proliferation, cancer cells have an incessant need of nucleotides for DNA synthesis. The folic acid (FA) is required for nucleotides synthesis, and in this view, Folate Receptor (FR) have acquired importance as target to image cancer proliferation by nuclear techniques [28–30]. In fact, the FR-α isoform is overexpressed in different human cancer tissues (such as ovary, lung, breast, kidney, brain, endometrium colon and hematopoietic cells of myelogenous origin; normal cells are instead very restricted in possessing folate receptor) [30], and it permits to folate analogue, such as the labeled probe, to enter the cells by endocytosis. Moreover, the labeling of receptor ligands doesn't affect their affinity for the receptor, and being small molecules, they have the great advantage to show a complete penetration of solid tumors and rapid clearance from FR-negative tissues [30].

In addition to PET and SPECT derivatives, folate was also labeled with near infra-red (NIR) fluorescent molecules, to perform fluorescence imaging (FLI) in preclinical setting. Among NIR fluorophores, the most used is indocyanine green (ICG), that in addition to its NIR fluorescent emission, it is the only dye that has been approved by the United States Food and Drug Administration for noninvasive NIR-fluorescence imaging for clinical application (to determine blood flow, to perform ophthalmic angiography, and to evaluate sentinel lymph nodes [31–32]). In order to study by FLI the expression of folate receptor (and as a consequence, the proliferation process), FA was conjugated with ICG in different designs [33–34]. In general, ICG-FA conjugates showed uniform accumulation in FR positive tumors and the probes were visualized until 24-48 hours. A background signal at the level of liver and urinary tract was also detectable only at early time points after probe injection, probably due to clearance of unbound probe.

Despite the wide medical application, ICG has several drawbacks (due to its physicochemical characteristics) that limit its use in the clinic [35–36]. In short, ICG is prone to aggregate and degrade in aqueous solution and has a short half-life even when stored in the dark (*t*_1/2_ = 16.8 ± 1.5 h at 22°C); it binds to plasma proteins when administered intravenously and has a blood circulation half-life of only 2-4 minutes; in addition, ICG doesn't permit an easy conjugation with proteins. In an effort to improve the stability and circulation half-life of ICG, it was enclosed into nanoparticles (NPs) [36], such as FA-ICG-PLGA-lipid NPs. When injected, these NPs localized only in tumor masses, whereas un-targeted probes (such as ICG-PLGA-lipid NPs) or ICG alone primarily localized at intestine level. After 24 hours, *in vivo* fluorescent signal was detectable only in tumors of FA-ICG-PLGA-lipid NPs injected mice. Moreover, several nanoparticles targeting FR were developed [37–40] using different fluorophores, and providing similar results.

Despite the availability of these probes, 2DG is used as surrogate marker for both glucose metabolism and for proliferation, since an increase in cell number is correlated with an increase of glucose consumption.

### Hypoxia

Another crucial process for many aspects of tumor development and growth is hypoxia. It has been indicated as a negative prognostic biomarker involved in chemo and radio resistance, and in tumor progression, as well as in the sustainment of the stem cell niche [41]. The key player of cell response to hypoxia is the hypoxia-inducible factor (HIF) [42–43], that induces the expression of different genes involved in neo-angiogenesis, vascular modelling, invasiveness, genomic instability and resistance to apoptosis, resulting in malignancy increase and reduction of chemo and radio treatment efficacy [44].

A recent and promising smart probe has been developed by PerkinElmer to study in an easy manner hypoxia in solid tumors and is commercially available: the HypoxiSense680. It is a Carbonic Anhydrase IX (CAIX) targeted fluorescent molecule used to visualize CAIX overexpression in tumors in response to regional tumor hypoxia [45–46]. The use of HypoxiSense680 allows the non-invasive imaging and the quantification of tumor sub-regions undergoing hypoxia-related changes. HypoxiSense680 clears from the bloodstream quickly, with a half-life of approximately 12 hours, whereas at the tumor site it accumulates within hypoxic regions with a half-life of 6h. Tumor hypoxia can be detected as early as 3h post-injection, with optimal signal to noise ratio measured at 12-24h, once the circulating agent has completely been cleared. In a recently published work by our research group, we used HypoxiSense680 as a biomarker of HIF-1 activity after treatment with Temozolomide (TMZ) in a mouse model of human glioma (U251 cell line). Cells were expressing Luciferase under control of HIF-1 activity and mCherry under a constitutive promoter. Obtained results demonstrated a correlation between HIF-1 activity and probe uptake by the tumor as expected since the presence in the CAIX promoter of Hypoxia Responsive Elements. These promoter sequences are recognized and bound by activated, nuclear HIF-1, inducing the transcription and further translation of the CAIX protein [47] as one of its targets. Our data, based on the use of this probe, showed a reduction of HIF-1 activity after TMZ treatment of the glioma model. In this context, also HypoxiSense680 uptake was statistically reduced 2 days after the beginning of TMZ treatment, and this reduction was strongly maintained after 1 week. These data correlated with Luciferase signal, showing a reduction of HIF1-driven Luciferase expression soon after 2 days of treatment; conversely, the constitutively expressed mCherry reporter, used as indicator of viability, showed a signal reduction only one week after the beginning of the treatment. Also immunohistochemistry (IHC) analysis showed that CAIX protein expression was decreased in glioma tissues in TMZ treated mice validating non-invasive imaging results. Additionally, IHC documented a concomitant delocalization of HIF-1 from the nucleus to the cytoplasm, confirming transcriptional regulation described by Optical imaging technique and the relation between HIF-1 transcriptional activity and CAIX expression. In this context, the identification of CAIX as a non-invasive quantitative biomarker of HIF-1 activity would support timely monitoring of tumor response to treatment. Furthermore, the use of this glioma model (U251-HRE-mCherry) would enable the *in vivo* assessment of novel specific CAIX probes for translational imaging procedures, making CAIX a promising theranostic imaging biomarker.

Recently, the feasibility was studied to use dual-emissive materials to combine fluorescence and phosphorescence to detect oxygen level and, as a consequence, hypoxia phenomenon. The first *in vitro* study was carried out on a cyanine dye conjugated with an O_2_-sensitive Pt porphyrin phosphor in a sol-gel matrix [48]. The possibility to combine a single component dye-polymer with both fluorescence and phosphorescence offers some advantages over the classical three mixtures (dye, ligand and linker), and among all, the stoichiometric rate between fluorophore and phosphor provides sample homogeneity and minimal dye leaching. The iodide-substituted difluoroboron dibenzoylmethane-poly-lactic acid (BF2dbm(I)PLA) sensor material holds these characteristics. Fluorescence emission depends only on molecular weight of the probe: high molecular weight samples are characterized by blue shifted emission, while low molecular weight samples show red shifted fluorescence. Conversely, phosphorescence is present at different level relative to O_2_ concentration. The probe was injected in a mouse bearing 4T1 mammary carcinoma [49], and the differences in phosphorescence emission was recorded by intravital microscopy imaging, after changing O_2_ concentration in breathing gas. The probe showed excellent contrast between the vessels and the tumor mass, with an increase in phosphorescence emission in presence of high level of O_2_.

### Angiogenesis

Angiogenesis is defined as the formation of new vessels from pre-existing vasculature and in tumor progression it is a fundamental process for the local expansion of tumor colonies. When tumor increases its size, the diffusion of nutrient and oxygen in tumor mass is no longer efficient and new vessels are formed to supply this deficiency (e.g. tumor hypoxia, HIF-1α nuclear expression levels and de novo angiogenesis are recognized interconnected events in cancer). Being a hallmark of cancer, the possibility to detect angiogenesis could help in the diagnosis and in the planning of cancer therapy [50].

Angiogenesis is also a key process for metastasis formation, because it determines the formation of fenestrated vessels that facilitate the spreading of tumor cells to distant sites. As a consequence, angiogenesis could be considered as an additional marker for metastatic potential.

Two targets for angiogenesis have been successfully imaged in humans and mice: the VEGF and its receptor and integrin α_v_β_3_.

#### VEGF and its receptor

Vascular endothelial growth factor (VEGF) [51] is a glycoprotein that acts as key mediator of angiogenesis induction: it binds to VEGF receptor 1 and 2, which are expressed on vascular endothelial cells, and normally are involved in embryonic development and wound healing. In cancer cells, VEGF production is up-regulated by mutation in oncogenes, hypoxia establishment and growth factor over-activation, resulting in the “angiogenic switch” [51], characterized by new vasculature formation and exponential growth of the tumor. The new vessels are structurally and functionally abnormal: they are irregular, twisted, hemorrhagic and not organized, having also dead ends. The result is a sub-optimal blood flow that increases hypoxia which determines further VEGF production, validating VEGF as a target for therapy.

Human VEGF has been conjugated with NIR fluorescent dye Cy5.5 (Cy5.5-VEGF), yielding a unique functionally active probe for *in vivo* molecular imaging by FLI. This molecule has been tested and validated in a subcutaneous murine model of mammary tumor [52].

Development of a mAb-based dual functional imaging agent, containing both NIR fluorescent molecules and a PET radionuclide, could facilitate the translation of FLI imaging into the clinical setting, providing synergistic advantages over a single imaging modality. For example, 1,4,7,10-tetraazacyclododecane-1,4,7,10-tetraacetic acid (DOTA) chelator was conjugated with amine-functionalized semiconductor quantum dots (QDs) and VEGF protein and was further labeled with ^64^Cu (^64^Cu-labeled DOTA-QD645-VEGF) [53] permitting to carry out fluorescence and PET imaging. Both NIR fluorescence imaging and microPET showed VEGFR-specific delivery of conjugated DOTA-QD-VEGF nanoparticles; good correlation was also observed between the images and *ex vivo* PET and NIR fluorescence organ imaging.

Changing strategy, Bevacizumab, a monoclonal antibody able to detect VEGF, was labeled with IRDye800CV, a near infra-red fluorophore (Bevacizumab-IRD800), and tumor uptake was evaluated in human xenograft-bearing athymic mice (injected with ovarian cancer cell line A2780, expressing high level of VEGF) during 1 week after tracer injection [54]. Images showed high signal- to- noise ratio in the tumors at day 2, and it remained constant until day 6. Given its specificity, it was also tested in an intraoperative setting in two models: a primary ovarian cancer and an intraperitoneal metastasis model of gastric cancer. In both studies the probe allowed to visualize tumor mass or metastasis. In the latter case the peritoneal cavity was open, whereas the primary ovarian tumor was detectable in spite of the presence of intact skin overlying the tumor. These findings strongly support future clinical application of NIR fluorescence-labeled tumor-specific antibodies in a wide range of clinical applications, including intraoperative image-guided procedures.

### Integrin

Integrin is a family of cell adhesion molecules that are involved in a wide range of interaction between cells and extra-cellular matrix. For example, the isoform α_v_β_3_ is differently expressed in new-born vessels and in tumor cells compared to normal tissues and endothelial cells, making it eligible for tumor imaging as well as for anti-neoplastic treatment [55]. This integrin strongly correlates with tumor progression and invasion in a variety of neoplasms (such as glioma, breast, ovarian and prostate cancers and melanoma), facilitating the spreading of cancer cells through blood vessel. The possibility to investigate α_v_β_3_ biodistribution could be a reliable method to study the relation between endothelium and tumors, since angiogenetic switch is correlated with endothelial cells and with their tube-forming capacity.

Integrin α_v_β_3_ binds to a wide range of extracellular matrix proteins (e.g. vitronectin, fibrinogen, laminin, collagen) characterized by an Arg-Gly-Asp (RGD) triple-peptide motif, and different probes based on RGD domains starting from linear [56] to cyclic [57–58] (both available from LI-COR Biotechnology - GmbH, Bad Homburg, Germany), to multiple peptides [59–61] were developed to improve the targeting capability and its affinity, to prolong the circulation time and to decrease the tumor washout rate. These peptides were labeled with different fluorescent molecules in order to *in vivo* study α_v_β_3_ biodistribution in tumors (see Table 2 for a full list of the different strategies for probe development).

**Table 2:** List of RGD-based probes

	RGD motif	Fluorophore	Other label	Tested in	Reference
**Cypate-linear-RGD**	linear	Cypate		A549 lung adenocarcinoma	[56]
**IRD800-linear-RGD**	linear	IRD800		U87MG, RCAS-PDGF and TS543 glioblastoma	[58]
**IRD800-cyclic-RGD**	cyclic	IRD800		U87MG, RCAS-PDGF and TS543 glioblastoma	[58]
**Cy5.5-cyclicRGD**	cyclic	Cy5.5		U87MG glioblastoma	[57]
**CLIO-Cy5.5-cRGD**	cyclic	Cy5.5	CLIO	BT20 mammary carcinoma	[64]
**QD705-RGD**	cyclic	QD		U87MG glioblastoma	[67]
^111^**In-DTPA-IRD800-RGD**	cyclic	IRD800	^111^In	M21 melanoma	[68]
**Integrin Trace**	dimer	ICG			www.intrace-medical.com
**Cy7-tetrameric RGD**	tetrameric	Cy7		U87MG glioblastoma	[59]
**RAFT-RGD-Cy5.5-SS-Q**	tetrameric	Cy5.5		IGROV-1 ovarian cancer	[60, 61]
^64^**Cu-DOTA-Cy5.5-RGD-knottin**	knottin	Cy5.5	^64^Cu	MDA-MB-435 mammary carcinoma and U87MG glioblastoma	[69]

For example, through a RAFT platform, capable of building scaffold molecules [62], a cyclic decapeptide forming a ring with two faces was generated. The upper face was used to link four copies of the RGD peptide, and the bottom face was conjugated with the Cy5.5 fluorophore and a quencher, making the probe un-fluorescent until the reduction of the disulfide bond (SS) during integrin mediated internalization, thus providing higher signal specificity. The probe was called RAFT-RGD-Cy5.5-SS-Q and was tested in IGROV-1 ovarian cancer models [60–61], showing good tumor contrast from 3 to 24 hours, with a peak at 10 hours.

The use of small, structurally simple RGD peptide-based tracers has some disadvantages. They are characterized by low tumor uptake due to the rapid tumor washout depending upon the sub-optimal receptor-binding affinity and the inadequate contact with cell-surface integrin receptors [63]. Even if dimeric RGD peptide tracers had some advantages over linear RGD, their tumor accumulation resulted not to be optimal as well. Conversely, the development of tetrameric tracers has resulted in high receptor-binding affinity, due to the higher molecular size of these tracers, determining longer blood circulation time responsible for the prolonged tumor retention [63].

Cy5.5-cyclicRGD was also linked to CLIO nanoparticles (containing an icosahedral core of superparamagnetic crystalline Fe_3_O_4_ -magnetite-), CLIO-Cy5.5-cRGD [64], forming a magneto-fluorescent nanoparticle for multimodal imaging of α_v_β_3_ integrin. Nanoparticles used as targeted agents offer the advantage of signal amplification by being able to be loaded with several molecules of both peptide and fluorophore [65–66]. Moreover, they have the advantage of more efficient targeting of the α_v_β_3_ integrin expressed in tumor endothelium (neo-vascularization) and of selective delivery of drugs or other molecules *in loco*. Probe uptake was assessed in mice implanted with mammary carcinoma cell line BT20. When tumor reached 3-4 mm of diameter, mice were injected with CLIO-Cy5.5-cRGD or with CLIO-Cy3.5-linearRGD, as control. Both compounds showed high fluorescent signal in liver and spleen, but only the cyclicRGD containing nanoparticles showed high specific signal in BT20 cells. Since the half-life of the molecule was 180 minutes, fluorescence imaging at early time point showed the presence of nanoparticles in the vasculature, while after 1500 minute, fluorescent signal reflected the specific accumulation at the tumor site, with a different enhancement for deep tumor slices. Also MRI images demonstrated a T2 signal at tumor level. The CLIO-Cy5.5-cRGD nanoparticles were readily detectable by fluorescence-based imaging and MR-based imaging at a dose of 3 mg/kg Fe, which is compatible with human use.

RGD peptides were also used to functionalize Quantum Dots (QDs) made of cadmium selenide cores overcoated with a layer of ZnS (which emits at 705 nm, and represents the best fluorophore among all QDs, even if its toxicity has not yet been well defined), and it has been called QD705-RGD [67].

**Figure 3:**
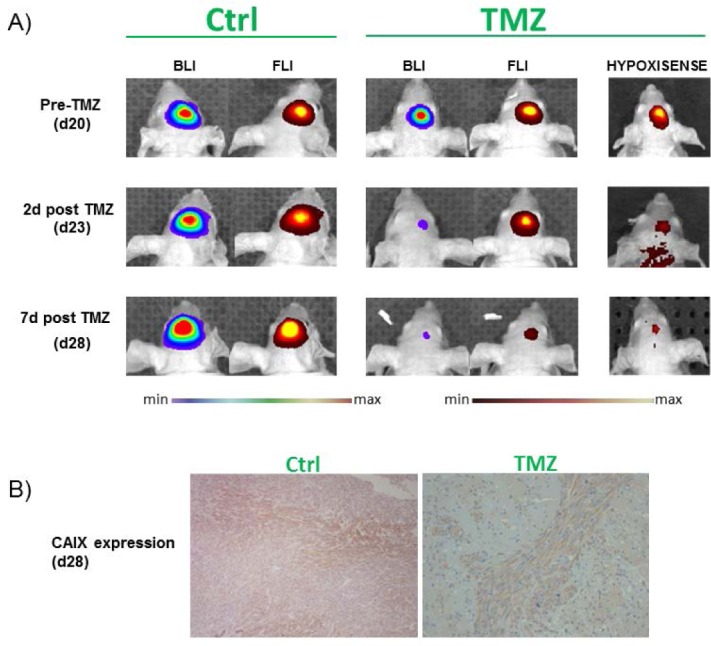
*In vivo* detection of Luciferase activity in orthotopic U251-HRE-mCherry glioma models and *ex vivo* validation **A.** 2-D luminescence (rainbow scale) and fluorescence (red-yellow scale) images of U251-HRE-mCherry tumors in control and TMZ-treated mice at different time points. The last column is the representative 2-D images of TMZ treated mice injected with HypoxiSense680 at different time points during TMZ treatment. Images are presented with the same scale bar. **B.** *Ex vivo* immunohistochemical staining CAIX in control and treated mice. TMZ=Temozolomide; CAIX=Carbonic Anhydrase IX.

Furthermore, RGD peptides were also labeled with IRD800 fluorochrome and ^111^In (^111^In-DTPA-IRD800-RGD [68]) or with Cy5.5 and ^64^Cu (^64^Cu-DOTA-Cy5.5-RGD-knottin [69], in which RGD was grafted into knottin [70], a cysteine knot peptide, identified from a library of genetically modified knottin peptides able to target α_v_β_3_, α_v_β_5_ and α_v_β_1_ integrins), in order to perform a multimodal analysis of integrin expression by optical and nuclear imaging.

Lastly, a non-peptidic probe capable of targeting α_v_β_3_ integrin in neo-vasculature (Perkin Elmer), called Integrisense [71], is commercially available and has been labeled with NIR emitting (645, 680 or 750 nm) fluorophores. Integrisense680 was injected in mice bearing either α_v_β_3_-positive tumor (HCT116 cell line derived form a colorectal carcinoma or A673 derived from an Ewing's sarcoma) or α_v_β_3_-negative but α_v_β_5_-positive tumor (HT29 - colorectal adenocarcinoma): imaging showed a stronger signal in α_v_β_3_-positive tumors, compared to HT29 α_v_β_3_-negative tumors, 4-24 hours post-injection. This difference in accumulation was ascribed to the expression of different integrins. When an excess of un-labeled parental compound was injected, a reduction in accumulation of Integrisense680 in α_v_β_3_-positive tumor was observed; moreover, after treatment with Avastin (a humanized anti-VEGF monoclonal antibody -bevacizumab- able to inhibit growth of rhabdomyosarcoma in mice) the Integrisense680 uptake significantly dropped down. These findings validated the specificity of the signal obtained with this probe, and its high affinity for the target.

In addition to the use of α_v_β_3_ integrin as molecular target for the study of neoangiogenesis, other tracers were developed targeting α_2_, α_3_ or α_4_ integrin by conjugation of specific peptides with Cy5.5 or AlexaFluor680 molecules [72–76] and these probes were visualized in different murine models of prostate, ovarian and breast cancer and lymphoma by optical imaging.

**Figure 4:**
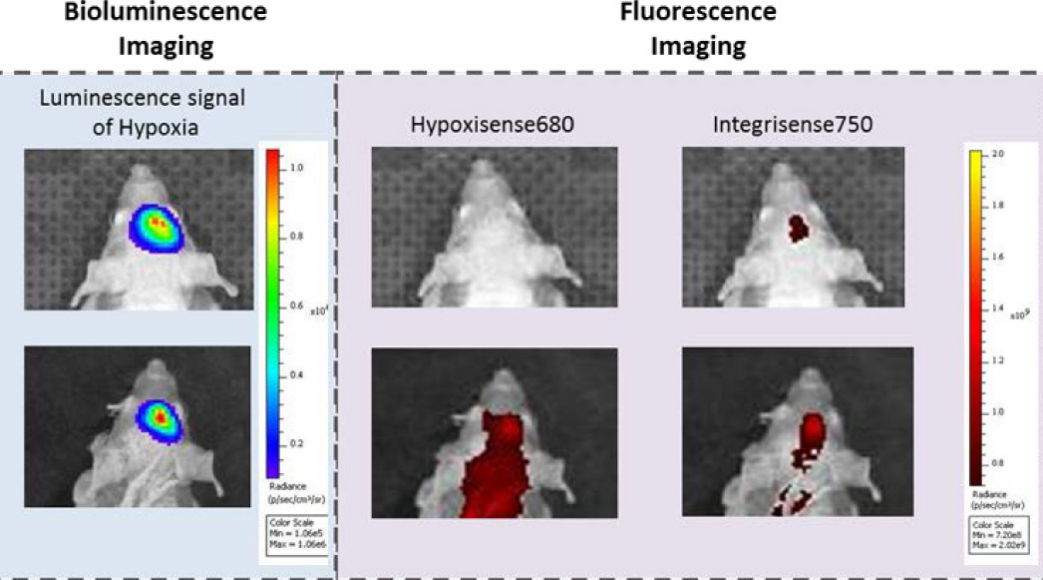
Optical imaging assessment of hypoxia and neoangiogenesis levels Mice were injected with an engineered glioblastoma line expressing Luciferase under the control of a HIF-1 inducible promoter. Images represent HIF-1 activity (Luciferase activity), hypoxia establishment (Hypoxisense680) and neo-angiogenesis (Integrisense750) during time, and show an increase of both hypoxia and angiogenesis deeply related to HIF-1 activity.

Since angiogenesis and hypoxia are strictly related within the context of tumor growth and progression (angiogenic switch), we started studying these two events by using specific fluorescent probes in a mouse model of glioblastoma [45–46] (Figure 4). This tumor cell line has been engineered to express Luciferase reporter gene under control of HRE (Hypoxia Related Element) sequence, as described above in paragraph 2.3, becoming a biomarker for HIF-1 activity also in the context of hypoxia establishment. This model has been used to assess the ability of Hypoxisense680 and Integrisense750 to monitor hypoxia establishment and neoangiogenesis, respectively, in relation to tumor growth. Luciferase activity has been used as internal control of HIF-1 activity involved in hypoxia establishment as already demonstrated elsewhere [45]. Figure 4 shows that HIF-1 activity increased with tumor growth during time and Hypoxisense680 confirmed hypoxia establishment as indicated by Luciferase activity increase. Consequently, as can be expected, Integrisense750 probe signal also incremented in relation to neoangiogenesis within the tumor lesion, probably as a consequence of HIF-1 activity increase in hypoxic regions and its effect on specific target genes involved in this process.

From the technical standpoint, this example is very interesting because it demonstrates the possibility of monitoring two different tumor aspects (hypoxia and neoangiogenesis) by using two different probes co-injected in the same mouse model. The different wavelength of the probes allows to distinguish (un-mix) the two different signals obtaining two image sets each specific to a different wavelength. Images can be also shown as a single image set to assess the co-localization of the two fluorophores. In this example, moreover, the possibility to assess HIF-1 activity also by measuring Luciferase activity allowed to use it as an internal control of tumor viability and, since day 18 after injection, of hypoxia establishment. Luminescence can be used to compare the signal obtained with the fluorescent probes, allowing to characterize newly produced probes, in view of their optimization.

### Invasiveness

Invasion entails the dissemination of tumor cells in the local microenvironment, whereas the term metastasis refers to the dissemination of tumor cells to distant organs through lymphatic or blood vessels; these are the most insidious and life-threatening aspects of cancer, and constitute the evidence of increasing tumor malignancy. Invasiveness and metastatic potential are supported by the up-regulation of specific proteases, such as cathepsins and metalloproteinases, which facilitate tumor cell invasion in the extracellular matrix [77]. The involvement of these proteins in the invasion and metastatic process highlights the importance of protease imaging for the assessment of tumor grading and the estimation of its metastatic potential in relation to treatment set up and evaluation as well.

#### Cathepsins

Cathepsins are proteases involved in different step of invasiveness [78]: first, they are involved in the degradation of vascular basement membrane during angiogenesis; second, they are implicated in the dissolution of cell-cell junctions and degradation of both the epithelial basement membrane and the extracellular matrix to allow cancer cells to spread from the primary tumor mass; third, intravasation of cancer cells into the blood or lymphatic circulation, and their extravasation in distant organs require the activity of these proteases.

In particular, cysteine cathepsins are proteolytic enzymes, consisting of 11 members: cysteine cathepsin B, C, F, H, K, L, O, S, V, W and X. They play an important role in cancer processes, and surveys in human tumors have suggested an association of certain cathepsins (especially B and L) with malignancy [79].

With the aim of studying the activity of these enzymes, activatable probes have been developed and called activity-based probes (ABPs). These probes are usually formed by three components: a reactive functional group known as a warhead for the specific enzyme targeting (in this case, the cathepsin), a linker for preventing steric congestions, and a reporter tag for visualization of the probe localization [80–82]. The possibility of using fluorescent molecules to label the probe opens the possibility to *in vivo* study the activity of cathepsins by optical imaging.

GB137 [83] is an example of this kind of probes, and was developed by using acylomethil ketone (AMOK) as warhead for enzyme activity detection (other probes were developed by linking fluorophores to different polymers for detection of cathepsin B [84] or K [85]). In this probe, the fluorophore Cy5.5 and the quencher QAY21 are linked to the structure, and the reaction between cathepsin B and L and AMOK determines the cleavage of the interaction between the quencher and the fluorophore, which becomes capable of emitting fluorescence. This probe was intravenously injected in breast cancer bearing mice, and the non-invasive, whole-body imaging allowed the direct monitoring of cathepsin activity in tumor and spleen, kidneys and liver (all organs where cathepsins B and L were expressed), and showed no background since the earliest time point (conversely, the use of the same probe without the quencher resulted in a general background until 12 hours after injection). The specific signal in tumor increased over time, reaching a peak at 6-8 hours post-injection. To validate the specificity of this signal, a blocking study was performed using K11777, an inhibitor that blocks the active sites of all cysteine cathepsins, showing a decrease of fluorescent signal of 60-80% in treated animals respect to controls [83]. These results suggested NIRF-ABPs as potentially valuable new imaging agents and powerful tools for preclinical testing of therapeutic agents *in vivo.*

An easy and powerful way to study the cathepsin modulation is the use of ProSense680 fluorescent probe, already commercially available (PerkinElmer) [86–87]. It is a protease-activatable fluorescent agent useful for *in vivo* studies, which is activated by proteases such as Cathepsin B, L, S and Plasmin, and it is also scheduled to enter clinical trials in the coming years for the detection of ovarian and colorectal cancer. ProSense is optically silent in its un-activated state and becomes highly fluorescent following protease-mediated activation. This probe was injected in mice bearing different types of tumors (such as ovarian [88] and hypopharyngeal cancer [87]), and provided a strong correlation among fluorescent signal of ProSense680, cathepsin activity and tumor size. Moreover, the Prosense680 was visualized with laparoscopic microscopy [86] in ovarian cancer in mice and *ex vivo* in human explanted tissues. This approach may be translated into the clinical setting for endoscopic high resolution detection of initial ovarian cancer, instead of classical biopsies [86].

Another important class of protease in cancer is the serine proteases, and in tumor, cathepsin D and E have gained a great importance.

For example, cathepsin E is a tumor associated intracellular non-lysosomal aspartic proteolytic enzyme, and its expression was observed in several tumors. cathepsins E was labeled with a NIR fluorochrome [89] (Cy5.5-DPGC-CathE) combined with a quencher (DPGC). The probe was injected in cathepsins E-positive MPanc96-E human tumor bearing mice and the probe localization was followed by fluorescence imaging. Images showed a clear signal at tumor site starting 24 hours after probe administration. The signal increased up to 72 hours, and tumor border visualization became very clear. A high fluorescent signal was also present in the upper quadrant of abdominal cavity, due to the liver ability of retaining molecules with high molecular weight, including the probe, and due to its high enzymatic physiological activity, including also cathepsin activity, which induced probe activation.

In summary, due to the central role of cathepsin in tumor progression, their visualization represents a valid target for tumor identification and/or for evaluating treatment effect.

#### Matrix metalloproteases

Matrix metalloproteases (MMPs) are a family of neutral metalloenzymes secreted as latent pro-enzymes that could be used as target for the metastatic process. MMPs require activation through proteolytic cleavage of the amino-terminal domain, and their activity depends on the presence of cofactors such as Zn^2+^ and Ca^2+^. Increase in MMP activity has been found to be correlated with invasion and metastatic potential in a wide range of cancers [90], and in this view, the possibility to non-invasively image MMP activity becomes an important theragnostic biomarker of tumor behavior. Two different types of probes were developed for non-invasive assessment of their activity through Fluorescence imaging: the first group is constituted by specific target molecules for different MMPs, conjugated with a fluorophore or also a quencher, the second group by MMP specific inhibitors.

In the category of target peptides, among the commercially available activatable probes, there is a FAST (Fluorescent Activatable Sensor Technology) probe, called MMPSense (PerkinElmer) [87]. It is a MMP activatable agent that is optically silent upon injection and produces fluorescent signal (due to the conjugation to 645-, 680- or 750-fluorochrome) after cleavage by MMPs. Activation can occur by a broad range of MMPs, including MMP 2, 3, 7, 9, 12, and 13. This activatable architecture offers high target specific signal with reduced background even if, in some cases, the substrate can be cleaved by other enzymes making the reaction, and the imaging data, non-specific. To describe MMPSense680 specificity, it was injected in subcutaneous hypo-pharyngeal squamous cell carcinoma bearing mice. A clear signal localized in the tumor edge (where metalloproteinase activity was expected) was detectable, whereas signal due to 2DG uptake, used to detect metabolically active tumor mass, was predominantly located at the core of this ring. *Ex vivo* histology showed that MMPsense fluorescent signal and MMPs (detected by immunohistochemical approach) co-localized in the tumor directly surrounding tissue (also named “invasive tumor border”), where MMPs are involved in the extracellular matrix degradation for tumor invasion.

Another probe that can be used for the MMP activity imaging is the MT1-MMP fluorogenic probe. It is composed by a NIR dye (Cy5.5), a MMP substrate, and a quencher (BHQ-3) [91]. It has been reported that MT1-MMP is retained for a long time in melanoma tumor cells and strong fluorescent signal in tumors is due to the action of different types of MMPs, such as MMP-2 and MMP-9. Its specificity in enzyme targeting has been demonstrated, since the injection of MMP-I, an inhibitor of different MMPs, produced a strong reduction in probe signal.

A MMP2 specific probe was developed to distinguish cancer foci (delineating the primary tumor in malignant glioma, medulloblastoma, prostate and intestinal cancer, sarcoma, as well as metastatic cancer foci) from adjacent normal tissues: the chlorotoxin:Cy5.5 (CTX:Cy5.5) [92]. This probe is based on the use of chlorotoxin (CTX) [93], that was demonstrated to show a preferential targeting for cancer cells [94–95]. For optical imaging, CTX was labeled with Cy5.5: this molecule is able to bind to a lipid raft-anchored complex that contains MMP-2 [96], and it is internalized within the cells [97]. In this paper, authors showed specific probe accumulation in the cerebellum of a transgenic mouse model of medulloblastoma, and CTX:Cy5.5 signal was also detected in primary tumors and metastasis during intra-operative surgery carried out in prostate and intestinal cancer, and sarcoma bearing mice. The imaging data were validated by IHC analyses, showing that portions of tissues characterized by Cy5.5 signal resulted to be cancerous, whereas adjacent non-fluorescent tissues were normal. This exquisite resolution and the safety of CTX-based probe make the use of this probe conceivable for pre-clinical investigation and a possible future translation in the clinical setting (at least for the superficial lesions).

MMP-2 (gelatinase, found to be correlated to numerous cancer types and to poor outcome) was also targeted by direct conjugation of Cy5.5 or rhodamine with its specific target peptide (MMP-Cy5.5 [98] or MMP2-TMR, respectively) [99], and probe accumulation was monitored during time, showing the specificity of the probe and identifying a sensitive method for the *in vivo* imaging of MMP-2 expression. Other probes were developed to target the specific activity of other MMPs, such as MMP-7 and MMP-13, by labeling their specific target peptides with Cy5.5 (MMP7-Cy5.5 [100] and MMP13-Cy5.5 [101], respectively).

Opposite to this strategy, some probes such as MMP2-TIMP-Cy5.5 [102] or AF489-Cy5.5 [103] were developed to image MMP activity by using fluorescent synthetic low-molecular-weight MMP inhibitors capable of targeting specific pockets of MMPs. For example, Cy5.5-AF489 was injected in mice bearing different tumors (A-673 Ewing's sarcoma, HT-1080 fibrosarcoma, MDA-MB 231 and BT20 breast cancer) and fluorescent signal was followed for up to 72 hours. Images showed different level of probe accumulation in different tumors, due to their different expression of MMP-2 and −9 (A-673 >> HT-1080 > MDA-MB 231>> BT20 cell line), and A-673 tumors showed the higher signal. Time course imaging showed a clear and fast Cy5.5-AF489 signal already after 30-45 min. While signal in non-tumor tissues remained constantly low, tumor masses showed a signal increase during time, and tumor to background ratio reached a maximum until 6 hours. After 24 hours, the signal intensity decreased for all tumors, and after 72 hours a still detectable signal was observed only in A-673 and HT-1080 tumors. Comparing the Cy5.5 signal with MMP expression, a clear correlation was observed. To confirm the specific targeting of this probe, when A-673 cell bearing mice were injected with parental un-labeled MMP inhibitor only a significantly lower signal, respect to non-treated tumors, was visualized. The probe was also compared with MMPsense750 in A-673 bearing mice, and both probes were able to detect MMP-positive tumors, with different time-windows: Cy5.5 signal was detected earlier in tumors (since 30 minutes, with a peak at 3 hours) than that of MMPSense750 (that showed a signal starting since 3 hours, with a peak at 24 hours). Thanks to small dimension of this kind of probes and their non-peptidic structure, the translation from pre-clinical to clinical studies might be possible.

### Inflammation

Inflammation is a protective response based on the intervention of immune cells, blood vessels and molecular mediators. As a rule it arises after microbial infection or trauma, with the aim to defend the host or to induce repair and regeneration of tissues, but it is also involved in the orchestration of tumor microenvironment, contributing to cancer cell survival, proliferation and migration [104–105]. For example, chronic inflammation is a pathological condition that can lead to dysplasia and cancer; besides, some tumor types, such as ovarian or gastric cancer, are related to a previous microbial infection, such as HPV-16 or Helicobacter pylori, respectively. In addition, infiltrating immune cells are usually observed in tumor lesions: some of these cells are related to a cytotoxic effector activity against tumor cells (e.g. CD8+ T cells, or Natural Killer cells) but other populations are involved in the stimulation of tumor progression (e.g. pro-tumoral macrophages M2); moreover, a gene expression profile correlated with inflammation was described in different tumors [104–105].

### To study inflammation in animal models, different optical probes were developed

Xenolight Rediject COX-2 probe is a commercially available targeted agent, (Perkin Elmer), able to specifically detect, by fluorescence imaging, Cyclooxygenase-2 (COX-2) activity in tumors. Its efficiency has been described in murine models of colitis-associated and sporadic colorectal cancer [106]. COX-2 is over-expressed in different kinds of tumors [107–108] and inflammatory tissues (at different levels), but not in normal cells. It is associated with all stages of cancer progression, and its amount increases with tumor malignancy [109]. It has been reported a strong correlation between COX-2 dependent fluorescent signal acquired by Optical imaging and subcellular COX-2 expression as assessed by IHC at the level of implanted intestine tumor [106]. Optical imaging of COX2 activity might help in the identification of patients that would benefit from aspirin treatment as a preventive strategy against colorectal cancer development or in the visualization and detection of pre-neoplastic lesions during surveillance endoscopy.

More recently, NANQ-IMC6 [110] probe was developed and permitted to study by fluorescence imaging the activity of COX-2 both in inflammation and cancers. This is an activatable probe in which the enzyme is necessary for the probe to become fluorescent. The probe is constituted by a chemical modification of COX-2 substrate indomethacin (IMC), which was linked to nitro-acenaphthenequinone (NANQ) fluorophore. The probe was injected in a murine model of sarcoma or in mice treated with carrageen to induce inflammation, and it has been shown that fluorescent emission is different between the two conditions, allowing to discriminate not only normal tissues from inflammation sites, but also different causes of inflammation. Based on these data, the use of this probe could be interesting to perform non-invasive differential diagnosis, or to guide, in real time (by using a hand-held ultraviolet lamp emitting at 365 nm), tumor resection during surgery.

Another example of probe for inflammation detection is the SLX-Lipo-Cy5.5 [111]. This probe is based on the use of Cy5.5 labeled Sialyl-Lewis^x^ carbohydrate, normally present on leucocyte surface, able to bind E-selectin. This molecule is expressed in inflamed tissues in response to Tumor Necrosis Factor α and Interleukin-1 that are released as a result of inflammatory stimuli. By targeting E-selectin it is possible to study the site of inflammation: experiments carried out in mice bearing Ehrlich ascites tumor cells showed SLX-Lipo-Cy5.5 signal at 24 hours post injection, with a peak at 48 hours (Lipo-Cy5.5 or G4GN-Lipo-Cy5.5 were used as controls, and no fluorescent signal was observed in tumor regions).

Alternately, macrophages have been labeled with a macrophage-specific fluorescent probe (MFP) [112]: MFP selectively allowed the visualization of monocytic and macrophagic cell populations *in vitro* as well as *in vivo*. This probe might be used to visualize sentinel lymph nodes during imaging-guided surgery, highlighting abnormalities related to inflammation and tumor infiltration in real time.

Another inflammation probe, used to monitor tumor invasiveness as well (see paragraph 2.5.1), is ProSense 750 probe. This probe has been used to evaluate inflammation associated to intestinal lesions in a mouse model of hereditary polyposis [113], reporting that probe activation correlated with the local density of pro-inflammatory cells infiltrating the lesion and with the amount of associated active enzymes at the tumor site.

A novel dual probe has been developed to study inflammation in a multimodal way by FLI and MRI (PFC-NIR dual-mode agent, PerFluoroCarbon-Cy5.5 probe) [114]. Breast carcinoma cell (4T1) bearing mice and naïve control mice were intravenously injected with the probe, and imaging was performed over time. The tumors were detectable in FLI images approximately six hours post-injection. No NIR signal was detected in the same region of control animals or on the contralateral flank of tumor bearing mice. A-specific NIR signal was also detected in the abdomen (liver and spleen), in both healthy and 4T1-derived tumor-bearing mice two hours post-probe administration. PFC has been also used to label macrophages [115–116], and it has been demonstrated that host phagocytes, recruited to the tumor microenvironment, represent the probe reservoir, rather than cancer cells. This has been confirmed by ^19^F MRI (7 Tesla). In fact, MRI images, thanks to the higher spatial resolution of this technique compared to optical imaging, revealed that inflammatory signal was located in the tumor periphery.

Another interesting strategy to study inflammation is the use of Xenolight Rediject Inflammation probe (PerkinElmer). This probe is based on the use of luminol as chemiluminescent source, enabling the detection of, mainly, acute inflammation largely mediated by tissue-infiltrating neutrophils, whose myeloperoxidase (MPO) activity is required for luminol bioluminescence [117]. Human HCT116 tumors were implanted in nude mice, and when tumor became visible, *in vivo* boosted splenocytes were intravenously injected into tumor-bearing mice. Bioluminescence imaging revealed inflammatory luminol bioluminescence 6 days after splenocyte transfer, showing neutrophil presence at tumor site. Mice were also injected with lucigenin [118], another bioluminescent probe for inflammation capable of being activated by macrophages: optical imaging displayed also lucigenin signal in the tumor. Control mice that did not receive conditioned splenocytes, showed no luminol or lucigenin signal at the tumor site. These two probes, by allowing non-invasive assessment of both neutrophil and macrophage-mediated inflammation, with no need of reporter gene expression, can be efficiently used in combination for the longitudinal *in vivo* evaluation of the inflammatory cascade in experimental models.

### Apoptosis

Apoptotic rate is another important tumor feature [119]. During tumor formation, mutation in oncogenes and alteration of cell processes may determine dysregulation of apoptosis reducing cell ability in inducing this pathway. On the other hand, tumor response to some anti-neoplastic treatments can result in an increase of the apoptotic cell rate [120]. Since the importance played by apoptosis (regulation and de-regulation) in tumor establishment, progression and treatment responsiveness, non-invasive apoptosis imaging provides important information for tumor characterization and management of the disease.

Different probes were developed in order to visualize apoptosis, mainly based on the detection of phospohatidilserine (PS) translocation in the outer membrane layer and on caspase-3 activity.

#### Phospohatidilserine

PS is a membrane glycoprotein, normally maintained in the inner leaflet of the plasma membrane by enzymes called flippases. In apoptotic cells flippases do not translocate PS, that can thus be found in the external layer of the cell membrane. Annexin V, a cellular protein which is known to have an anticoagulative function [121], is able to bind to PS, targeting apoptotic process [122].

AlexaFluor750 [123] (AnnexinV750-Vivo, Perkin Elmer), Cy5.5 [124], IRDye [125] and Quantum Dots (QDs) [126] were used to label Annexin V, sometimes also in presence of gadolinium [126]. By using these kinds of probes, fluorescence imaging showed minimal probe uptake in non-treated mice, with only a low a-specific signal detected in the kidneys. Following the treatment with pro-apoptotic drugs (such as doxorubicin, cetuximab or cyclophosphamide), a strong signal in tumors of treated mice was observed. The availability of radionuclide labeled Annexin V allows an easier potential translation of preclinical results in the clinical nuclear-based imaging setting.

Even if labeled Annexin V is the most used probe for apoptosis studies, there are some pitfalls in its use, such as non-specific accumulation in liver and kidneys, reduced affinity for PS after labeling, and exposition of PS during necrosis as well, producing a non-specific targeting of programmed death process [127].

#### Caspases

Caspases play a central role in the transduction of Death Receptor apoptotic signals [128]. Caspases are highly conserved cysteine-dependent aspartate-specific proteases. There are two types of caspases: initiator caspases (caspase 8, 10, 9, 2), and effector caspases (caspase 3, 7, 6). The activation of initiator caspases requires binding to specific oligomeric adaptor protein; effector caspases are then activated by these active initiator caspases through proteolytic cleavage. The active effector caspases then proteolytically degrade intracellular proteins to carry out the cell death program.

Caspase 3 is activated by interaction with caspase 8 and 9. The targeting of its activity is index of advanced apoptosis, and some probes have been developed to detect it.

An interesting probe for non-invasive fluorescence imaging of apoptosis based on Caspase 3 activity is the TCAPQ647 activable probe [129], in which the amino acid effector caspase recognition sequence, DEVD, was flanked by an AlexaFluor645 fluorochrome and a quencher (QSY21), and at its N-terminus was placed the amino acid permeation peptide sequence (RKKRRQRRRG), of the human immunodeficiency virus-1Tat (HIV-1Tat), necessary to allow the entry of the probe into the cells. *In vivo* characterization of this probe [130] showed an apoptosis correlated fluorescence emission in xenograft tumor models of colon cancer.

The tAB50-Cy5 and AB50-Cy5 probes [131] were labeled with Cy5.5 and developed to target both effector caspase 3 and 7, by using a common DEVD sequence, and a Tat or non-Tat peptide, respectively, for entering the cells. The caspase activation was monitored in xenografts of human colorectal cancer models in which the apoptosis was induced by treatment with the monoclonal antibody Apomab, a pro-apoptotic agonist of the Death Receptor 5, which causes apoptosis. Both probes permitted to detect the activity of both types of caspases in tumors, with different kinetics. The signal of tAB50-Cy5 probe was substantially brighter than that produced by the AB50-Cy5 probe, but its clearance was slower, resulting in a lower tumor to background ratio for AB50-Cy5 probe, at least at early time points. These data highlighted the possibility to noninvasively monitor apoptosis in tumors treated with chemotherapeutic agents using fluorescence based imaging strategies.

Another example of probe for the *in vivo* assessment of apoptosis through caspase activity targeting is the PPB probe [132]. PPB is formed by a ligand for folate receptor capable of targeting tumor cells, an infrared fluorochrome pyropheophorbide α (Pyro), a black hole quencher 3 (BHQ3), and a peptide linker (GDEVDGSGK) specific for caspase-3 (in fact it contains the tetrapeptide motif DEVD for caspase-3 recognition). Thanks to the use of Pyro, the probe combines therapeutic (photodynamic therapy) and imaging functions: Pyro is a nontoxic light-sensitive molecule capable of becoming toxic when exposed to light, producing singlet oxygen that disrupts the mitochondrial membrane [133–134], inducing in turn apoptosis (therapeutic function). This determines activation of caspase 3, able to cleave the probe and simultaneously to separate the fluorochrome from the quencher allowing the fluorescent signal emission and the identification of apoptotic cells (imaging function). To study *in vivo* the activity of this probe, mice were implanted with liver hepatocellular carcinoma cells and after 20 days, mice were injected with PPB [135]. Then tumors were illuminated with a laser scan at 670nm (at a light dose of 90 J/cm^2^ per tumor) 3 hours after probe administration. The light is able to activate Pyro, and to determine the induction of the complete cascade including caspase 3 activation and fluorescent signal emission in the area of PPB accumulation and PDT treatment. *Ex vivo* analysis showed that this area was highly apoptotic, validating the specificity and efficacy of this probe for both therapeutic and imaging strategies.

## TUMOR CELL BIOMARKERS

Many other tumor cell targets are used to characterize specific neoplasms, providing information about cellular features associated with a specific histotype and correlated with tumor responsiveness to treatments, its aggressiveness and a prognostic indication. With the continuous development of *in vivo* imaging procedures, an increasing number of tumor cell biomarker can now be non-invasively studied by using adequately labeled specific probes.

The non-invasive assessment of tumor biomarker can be of great importance in several steps, from diagnosis and staging, to biological characterization of the tumor, evaluation of the response to treatment and evaluation of residual disease, thus aiding in the prediction of clinical outcome and limiting the need for biological samples and invasive procedures (such as biopsies).

Non-invasive tumor cell biomarkers can include membrane or intracellular receptors for hormones and growth factors, adhesion molecules, enzymes and fetal or embryonic proteins.

### Oncofetal antigens

Some cancer types usually overexpress and secrete oncofetal antigens, such as carcinoembryonic antigen (CEA) or alpha-fetoprotein: these proteins participate in embryogenesis, and their production quickly declines after birth. However, high CEA levels or titers of antibodies to carcinoembryonic antigen (CEA) are significantly detectable in the majority of patients with colon, pancreatic and breast cancers. On the contrary, only approximately 5% of normal individuals have detectable CEA reactive antibody titers.

Regarding CEA expression [136], specific probes were developed to detect *in vivo* this oncofetal protein, by labeling specific monoclonal antibodies with Cy5.5 [137], Alexafluor488 [138], fluorescent cyanine DY-676 [139], QD820 [140], or bioluminescent proteins such as Renilla Luciferase [141] and Gaussia Luciferase [142]. Even if Renilla and Gaussia Luciferases are not fluorescent probes, this is an alternative strategy that can be used to target CEA by Optical imaging using Bioluminescence. Renilla Luciferase labeled probe (called Db-18-Rluc8) is formed by three components: a T84.66 diabody for targeting CEA positive cells, a Rluc8 enzyme (an isoform of Renilla Luciferase) for oxidation of coelenterazine substrate (necessary for light production), and a linker peptide of 18 amino acids to connect the two subunits. In small living animals, Db-18-Rluc8 localized in CEA-positive tumors and after injection of coelenterazine, a bioluminescent signal can be observed in neoplastic lesions thanks to the specific binding of the probe to tumor cells.

Alternatively, the targeting of alpha-fetoprotein (AFP) was carried out through monoclonal antibodies against AFP conjugated with Quantum Dots, AFP-QD590 [143], in order to study the accumulation and retention of AFP-QD at the site of xenografted hepatocellular cancer tumors, even if, to date, this probe was used only in *ex vivo* fluorescence imaging procedures.

### PSA

Prostate Specific Antigen (PSA) is the most used serum biomarker for prostate cancer screening and monitoring. However, its expression is not fully specific for oncological conditions, since it is present at high levels also in benign prostatic hyperplasia or in case of prostatitis. In normal tissues or in benign conditions, high concentrations of active PSA are stored in the prostatic collecting ducts [144]; in this setting only a small amount of enzyme can leak out, but it forms a complex with its inhibitor, the alpha-1 anti-chymotrypsin (ACT), generating an inactive PSA that can anyway be detected in serum samples by classical procedures. In cancer, this architecture is disrupted, and increased amount of PSA is accumulated into the tissue interstitium and is free to leak out in the serum where it is inactivated and detected. The higher level of PSA in the interstitium is related to an increased tumor growth rate, and can assume a causal role in prostate cancer progression [145].

Enzymatically active PSA is detectable only in prostatic tumor tissues, and can be considered a more specific target for the differential diagnosis of tumors from other pathologies. In this scenario, a specific probe for the active form of PSA has been developed and it is commercially available (Perkin Elmer): PSA 750 FAST [146] Fluorescent Imaging Agent. This is an optically silent probe in which the fluorescent signal appears only after the occurrence of an enzymatic cleavage by active PSA. It has been injected in mice bearing subcutaneous PSA positive or PSA negative tumors, showing a specific probe accumulation and fluorescence activation in PSA positive lesions, as validated in *ex vivo* sections. In fact, tumor regions that show extensive perfusion and vascular leakage do not show activation of PSA750 probe, because the combination with plasma components induces the formation of complex of PSA with ACT, resulting in ablation of enzyme activity, validating the specificity of this probe for the active form of PSA. PSA750 is a unique and powerful tool for preclinical prostate cancer research, and its future potential translation into the clinical setting could be of great benefit for transrectal or laparascopic prostatic imaging.

*In vivo* imaging of PSA has been based also on the use of PSA-specific antibodies labeled with ICG [147] or QDs [148–149] or PSA-specific small molecules used to bind PSA *in vivo* thanks to their more tractable pharmacokinetics and the more rapid washout from non-target sites, respect to antibodies. These molecules were labeled with IRD800CW, IRD800RS, Cy5.5, Cy7, or a derivative of indocyanine green (ICG) [150] to assess their accumulation by fluorescence small animal imaging.

### EGF/EGFR

**Figure 5:**
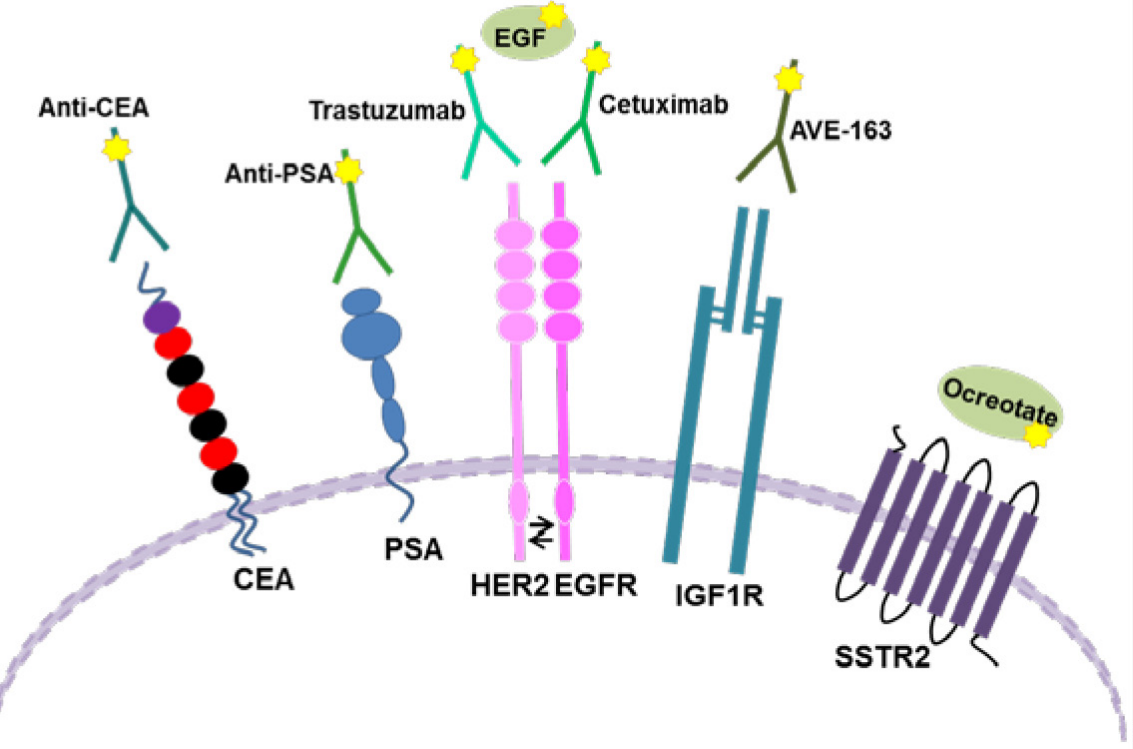
Schematic representation of different Tumor Cell Biomarkers The image shows the membrane localization of some molecules used as biomarkers, and the different possibilities to target them.

Epidermal growth factor (EGF) is a 53-amino-acid membrane protein able to stimulate the growth of epidermal and epithelial cells, and involved in cell proliferation, survival, adhesion, migration, and differentiation [151]. The EGF receptor family consists of four transmembrane receptors, including EGFR (HER1/erbB-1), HER2 (erbB-2/neu), HER3 (erbB-3), and HER4 (erbB-4) [152]. In oncology EGFR and HER-2 receptors have become very important as potential targets of biological specific drugs (Figure 5).

EGFR [151] (HER1) is located on cell surface and it is activated by different ligands, including epidermal growth factor and transforming growth factor α (TGFα). Upon activation EGFR becomes active and pairs with another member of its family, such as HER2, to create an activated heterodimer. The dimerization stimulates the activity of protein-tyrosine kinase. Mutations that lead to EGFR overexpression or constitutive activation have been associated with a number of cancers (e.g. it is up-regulated in 60-80% of colorectal cancer [153]), determining uncontrolled cell division and tumor proliferation, and they are correlated with poor prognosis.

Different fluorescent probes for EGFR targeting have been developed, and they are principally based on the use of anti EGFR antibodies labeled with different fluorophores. Cetuximab (or Erbitux, a monoclonal antibody already used in cancer therapy) has been labeled with Cy5.5 [154–156], QD840 [140] or Bodipy-FL [157] (a fluorophore that emits at 513 nm). Cy5.5-Cetuximab [156] has been injected in mice bearing MCF-7 and MDA-MB-231 breast cancers, and the tumor localization was monitored by fluorescence imaging. Images showed high probe accumulation at tumor site, while the administration of an excess of unlabeled Cetuximab showed a reduction on tumor contrast due to the competition with the labeled antibody for the specific target, even if a low background signal remained detectable.

However, the pharmacokinetics of intact mAbs showed high liver uptake and slow blood elimination [158] (as demonstrated with radiolabeled antibodies), confirming that whole Abs are generally not ideal for imaging procedures.

In this regard, a novel class of recombinant affinity ligands (Affibody molecules) for EGFR was constructed on the basis of a 58-amino-acid Z-domain residue deriving from the IgG-binding domain of staphylococcal protein A [159], and showing binding affinity to EGFR with Kd values of 25-50 nM. These affibodies were labeled with Cy5.5 [160–161], AlexaFluor680 [162] or IRD800 dye[161]. Affibodies labeled with IRD800, called Eaff800 [161], have been injected in a mouse model of human skin carcinoma, showing probe accumulation in tumor, kidneys and liver. The tumor signal was detected 1 hour post injection, and it increased after 1 day; subsequently, it decreased over time. The disappearing of signal was faster in non-tumor tissues, producing an increase in tumor to background ratio with a peak at 24 hours. In addition to the affibodies, single chain antibody fragments, consisting of the heavy and light variable chains of an antibody linked with a flexible peptide and characterized by a molecular weight five times smaller compared to native mAbs, but maintaining the same affinity [163–164], were also labeled with QD580 [165].

One of the advantages of fluorescence imaging is the possibility to image multiple specific targets at the same time, exploiting different probes labeled with distinct fluorophores. Several examples have been described: lung carcinoma bearing mice were co-injected with QD800-RGD, QD820-antiCEA and QD840-antiEGFR [140] and MultiSpectral Fluorescence Imaging (MSFI) allowed to differentially resolve the information given by the three probes. A fluorescent signal was observed for all the probes in primary and in metastatic lesions, as well as in lymphatic basin, as expected, since lung cancer can spread locally near the tumor or in the regional lymph nodes. In primary lesions, QD-antiCEA signal was lower than QD-RGD and QD-EGFR, correlating with their different expression in the lung tumor in which CEA is less expressed than the other two targets (α_v_β_3_ integrins and EGFR). These results have demonstrated that these QD bio-conjugates may be used as probes for the simultaneous detection of multiple tumor markers *in vivo*.

Since antibodies are approximately 25-fold larger than EGFR ligands, they may not be easily transported within solid tumors, and in a different imaging strategy EGF was labeled with Cy5.5 [166] and IRD800 [167–168], available from LI-COR Biotechnology - GmbH, Bad Homburg, Germany, to study EGFR density on tumor cells. The probe was tested in a breast cancer model, showing probe accumulation in EGFR positive tumors. Pre-treatment with Cetuximab prevented the labeled-EGF probe binding in the EGFR positive tumors, validating the specificity of the signal.

### ERBB2

The human epidermal growth factor receptor 2 (erbB2, HER2, or neu) [169] is an oncogene involved in the proliferation, migration, and invasion of cancer cells; for example it has been found to be amplified in 18-20% of breast cancers [170]. HER-2 positive breast cancers showed a worse prognosis compared to negative tumors, but the availability of targeted therapies constituted by monoclonal antibodies directed against HER-2 significantly improved the clinical outcome. In this setting, the detection of this biomarker on tumor cells is critical for the identification of the best treatment option.

In this context, trastuzumab (a humanized monoclonal IgG1 antibody, already approved for clinical use for anti-cancer therapies in both Europe and North America) was labeled with different fluorophores (Cy5.5 [171], AF750 [172–173], AF680 [174–175], RhodamineG [176–177], ICG [178] or QD800 [179]). Nowadays it is also commercially available as HER2sense, labeled with a red fluorophore emitting at 661 nm (Perkin Elmer [180]), allowing to *in vivo* study HER-2 expression mainly in mammary tumors. Activatable probes, constituted by Cy5.5 or Alexa680 labeled trastuzumab [175] bound to a quencher, have been injected in 3T3 sarcoma bearing mice. After binding to HER-2, the antibody was gradually internalized within HER-2 positive cells, and addressed to degradation into the lysosomes. Within these organelles, the quencher was separated from the fluorophore, and light was emitted. In this way, light emission resulted to be specific for HER-2 targeting. Both probes allowed the visualization of tumors, but Alexa680 labeled probe displayed a higher signal to noise ratio respect to the Cy5.5 labeled one. In fact, Cy5.5 resulted to be internalized also in liver, preventing the possibility to specifically target HER-2 positive tumors within this organ. Furthermore, it showed also a quote of a-specific binding to HER-2 negative tumors, probably due to its high lipophilicity, charge and molecular weight.

In order to ameliorate the unfavorable pharmacokinetics of whole antibodies, as described before, Affibody molecules for HER2 were also developed from one of the IgG-binding domains of staphylococcal protein A [181] (Kd values of <50 nM). In particular, the synthetic Affibody molecule His6-ZHER2:342-Cys (named ZHER2:342) was labeled with DyLight750 [182] and QD800 [183]. Moreover, it was fused to an Albumin Binding Domain (ABD) to further prolong the circulation half-life of the probe, and then was labeled with AlexaFluor750 [184] and DY-680 [185]. ABD-AF750-affibody, called ABD-ZHER2:342, was compared to AF750-labeled trastuzumab and with monomer or dimer affibodies, showing a strong signal in the mouse model around the human SKBR-3 breast tumor and in the kidney area, detected since 8 hours after injection (earlier respect to Trastuzumab, that localizes at tumor site 24 hours after injection), with a peak at 24 hours and a decrease after 72 hours (longer than monomeric and dimeric affibodies, that showed a weak signal only between 6 and 8 hours); moreover, the tumor to background ratio was higher for ABD-ZHER2:342, suggesting that ABD-fused ZHER2:342 could be used as a specific probe for a better non-invasive characterization of HER-2 expression *in vivo* [184]. Furthermore, the use of ZHER2:342-DyLight750 or AF750 labeled probes permitted an early characterization of HER2 expression, thanks to their relatively fast pharmacokinetics, allowing a precocious assessment of HER2 expression *in vivo* within a shorter observation time [182] and reducing the time window required in future clinic application.

In addition, HER-2 expression was targeted also by liposome labeled with QDs (named QD-IL) [186]. Anti-HER2 single chain F_v_ fragments (scF_v_) and QD800 were attached to the end of PEG chains bound to a liposomal core. The probe was intravenously injected in MCF-7 mammary tumors bearing mice and fluorescence imaging showed a strong signal at the tumor site (as well as in mononuclear phagocytic system organs where liposome were cleared). However, the targeted probe failed in showing any difference in tumor uptake from the non-targeted ones, probably for the enhanced permeability and retention (EPR) effect typical of tumors [187–188]. Imaging data were validated on *ex vivo* tumor sections, which showed extensive accumulation of the probe within tumor cells. These findings showed the important role played by liposomes in targeting tumors with the possibility to use them as drug delivery system with high efficiency, even if the targeting should be further optimized.

### IGFR1

The type I insulin-like growth factor receptor (IGF1R) is a tyrosine kinase receptor involved in cancer progression and metastasis formation [189–191] and its over-expression and activation has been reported in many cancer types, especially in breast cancer. Moreover, its expression is correlated with poor prognosis. Similarly to HER2, IGF1R expression monitoring is crucial for optimization of treatment efficacy. Different monoclonal antibodies are already used in phase I, II, and III clinical trials, but no data are available about the expression of IGF1R in primary tumors before the beginning of the treatment. Experiments carried out on xenograft mouse models showed down-regulation of the receptor after treatment with these antibodies, followed by a decrease in tumor growth [192–193]. For this reason, IGF1R down-regulation could be used as a biodynamic marker of antibody delivery and a potential indicator of treatment response.

In order to easily target the expression of IGF1R also in the metastasis, the IGF1R-specific antibody, AVE-1642, was conjugated to Cd/Te QD705 and with AlexaFluor680 [194], both with a peak emission at 705 nm, and probes were injected in R-/IGF1R cells bearing mice, in which IGFR1 is depleted, or in MCF-7 tumors bearing mice, whose tumors express high IGF1R level; probe accumulation was followed by fluorescence imaging. Mice injected with QDs-AVE-1642 showed fluorescent signal in liver, spleen, lymph nodes and bone marrow, all organs of the reticulo-endothelial system (RES) able to phagocyte nanoparticles, and are characterized by highly permeable sinusoids, which allow the extravasation of the probe, but no signal could be detected in tumors. *Ex vivo* analysis confirmed that no fluorescence could be detected in tumor masses, and also down-regulation of IGFR1 was not observed. In addition, mice were treated with clodronate, in order to deplete macrophages in RES, however no signal was detected at tumor level. On the contrary, mice injected with AlexaFluor680-labeled antibodies showed specific tumor signal from 1 to 4 days post-injection, whereupon signal decreased in the following 10 days (and no signal was detected in the abdominal area). *Ex vivo* data confirmed the imaging data, demonstrating also a down-regulation of the receptor. These findings suggest that antibodies labeled with small molecules efficiently allow to image IGFR1 expression and its down-regulation.

### SSTR2

Finally, Somatostatin Receptor, especially the subtype 2 (SSTR2), is another tumor cell marker found to be up-regulated in neuroendocrine (but also in some non-neuroendocrine, such as breast cancer and lymphoma) cancers [195]. Furthermore, it has been also found to be up-regulated in proliferative endothelial cells near tumor masses. For these reasons, it has been identified as a target for anti-cancer therapy.

In the clinic, the ^68^Ga-DOTATOC is routinely used for the differential diagnosis of neuroendocrine tumors. This probe is based on ocreotate, a stabilized analog of somatostatin labeled with ^68^Ga [196].

According to clinical procedures and with the aim to target this receptor for optical imaging, octreotate, was labeled with indotricarbocyanine [197] (ITCC) or with 5-carboxy-fluorescein [198] (emitting at 518 nm it was revealed by intravital microscopy imaging). ITCC-ocreotate was injected in mice bearing subcutaneously implanted RIN38 β-cells: fluorescence imaging showed an increase of the tumor to normal tissue ratio immediately after intravenous injection, and between 3 and 24 hours, the signal intensity in the tumor was three fold higher than in normal tissues. Other cells normally expressing somatostatin receptors were not detected during this procedure: for example, neuronal cells of the central nervous system did not show any signal, probably because the probe is not able to cross the blood/brain barrier, whereas neuroendocrine cells escape the visualization because they are dispersed within organs.

Since somatostatin receptors are over-expressed by the majority of gastric and breast cancers, the use of fluorescent labeled probes might help in the intraoperative identification of tumor lesions.

### GPCR

The G-Protein Coupled Receptors (GPCRs) form a big receptor family involved in the transduction of different kind of signals [199–200]. These receptors are formed by seven-transmembrane α-helices, and after binding with the specific ligand, they expose intracellular sites which interact with the G-protein heterotrimer containing α, β and γ subunits. This process determines the dissociation of bound GDP and the binding of GTP to α subunit, resulting in the dissociation of α-GTP and β γ subunits, which in turn are capable of activating different downstream pathways and effectors.

These receptors are embroiled in neurotransmission, hormone and enzyme release from endocrine and exocrine glands, immune response, muscle contraction and other physiological conditions. Recently they were found to be upregulated during tumorigenesis and linked to cancer progression and metastasis, by increasing proliferation and vascularization, evasion of immune surveillance, and increasing microenvironment invasion and tumor cell dissemination. They are also correlated to chronic inflammation and tumor establishment. As a consequence, targeting of GPCR could provide information about cancer detection and therapy.

Even if there is a broad range of ligand for GPCR, only two mitogen acting through this class of receptor, have been labeled with AF680 or Cy5.5 to perform oncological imaging: gastrin and endothelin, respectively.

Gastrin [201] is well established as a growth factor for the gastro-intestinal tract and the pancreas, and acts through activation of MAP kinase pathway effectors, e.g. ERK1/2, JNK, p38-MAPK and STAT3. Its expression has been observed in different kinds of cancer, such as colon cancers and primary colorectal carcinomas. Moreover, it has been found upregulated in other solid tumors with endothelial origin: gastric, pancreatic lung and ovarian cancer [201]. By autocrine effect on GPCR, gastrin realizes its effect, and the over-expression is mainly due to the deregulation of different oncogenes affecting downstream the gastrin promoter activity.

Gastrin peptide was labeled with AF680 (AF680-gastrin) [202–203], and imaging was performed to detect breast and prostate cancer lesions. AF680-labeled probe was intravenously injected in immunodeficient T-47D breast cancer bearing mice [202] and fluorescence imaging was performed after 15 minutes. Images showed high probe localization at the tumor masses. When unlabeled gastrin peptide was injected before probe administration, only a little signal could be visible, showing the high specificity of the probe. In a second set of experiments, mice injected with PC3 cells were followed over time [203], and in addition to probe localization within primary tumor, AF680-gastrin-based probe permitted also the detection of micro-metastases on the retroperitoneal wall under the kidneys and in the lumbar region near aorta. MRI and the *ex vivo* validation by means of histopathology procedures confirmed that they were clusters of tumor foci with sizes of approximately 200-400 mm in diameter.

Endothelin 1 (ET-1) [204] acts, in physiological conditions, to constrict blood vessels and raise blood pressure. Its cognate Endothelin A receptor (ET_A_R), belonging to GPCR class, has been found to be upregulated in different kinds of solid tumors, including thyroid, ovarian, prostate, colon, breast, bladder and lung cancers and it is involved in survival and proliferation of cancer cells, in migration, stemness and therapy resistance. In fact, ET-1 induces the activation of phospholipase C, which in turn influences calcium metabolism and activates protein kinase C: this enzyme regulates the proliferation of surrounding cells through activation of MAPK pathways, affects the apoptosis (by altering phosphorylation pathways), and promotes neoangiogenesis (by increasing growth factor transcription, e.g. vascular endothelial growth factor). Starting from these considerations, the *in vivo* visualization of ET receptor expression would be interesting for the staging and evaluation of therapy response.

ET-1 peptide was labeled with Cy5.5 (Cy5.5-Endothelin) [205] and a biodistribution study was performed in healthy mice showing probe uptake in the lungs, followed by kidneys, liver, heart, spleen and muscles. The lowest signal was detected in the brain. After 24-48 hours, the signal strongly decreased in all organs except lungs, which showed only a modest reduction. Then, the probe was tested in an orthotopic model of papillary thyroid cancer (K1 cell line) [206], that expresses high level of ET_A_R, and showed high fluorescent signal localization at tumor level up to 48 hours after tracer administration. The specificity of probe targeting was demonstrated by using PD156707 as blocking agent: imaging showed a reduction in probe uptake, if injected after receptor blocker. Moreover, the probe was tested in subcutaneous xenograft models obtained using two mammary cell lines (BT-20 and MDA-MB-231), a fibrosarcoma cell line (HT-108) and the already used K1 cell line (thyroid cancer). *In vitro* ET_A_R was found to be expressed in HT-108 and MDA-MB-231 cells (beyond K1 cell line), whereas no expression was found in BT-20 tumors, which express ET_B_R. K1 and HT-108-derived tumors showed high probe uptake in the whole imaging period, whereas BT-20 and MDA-MB-231 did not show a specific uptake, and the fluorescent signal was similar to muscles.

These data support the implementation and the use of intraoperative or endoscopic imaging devices, particularly when clinical practice is easy to image: for example, its use in the fluorescence-guided thyroidectomy, similarly to the procedure used in neurosurgery.

## TRANSLATION INTO CLINIC

Recent improvements in technology and scientific knowledge have introduced the use of Near-Infrared Fluorescence Imaging into clinics, and in the last decades, fluorescence has been used to guide surgeons during oncologic interventions. Conventionally, the navigation was done using X-ray fluoroscopy or ultrasounds. However, even if these techniques provide anatomical information, they do not provide findings about a specific molecular process or target. Moreover, fluoroscopy exposes patient and medical staff to ionizing radiation, while ultrasounds require a direct contact with the tissue, with obvious problems in an intraoperative setting. Fluorescence Imaging offers the possibility to overcome these limits, providing good sensitivity and specificity.

Among all available fluorophores, ICG is the only near-infra red dye, emitting at 820 nm, approved by FDA (Food and Drug Administration) and EMA (European Medicines Agency) for use in humans. The current indications are the determination of cardiac output, hepatic function, liver blood flow and ophthalmic angiography. Moreover, it has been used for lymphatic system imaging, and its use was tested also for nodal staging in cancer and breast lesion characterization (discrimination between malignant and benign lesions) [207]. All these last applications are important in the oncology field, and will be discussed below.

Lymphatic System is difficult to visualize, because it cannot be cannulated, but alterations in lymphatic flux and function are sometimes associated to cancer metastasis and to lymphedema following nodal staging surgery. ICG allowed to visualize the direction of lymphatic flux, following intradermal administration, to the draining lymph node: in a healthy person, the flux is linear, and velocity or pump frequency can also be recorded. Conversely, lymphatic system visualization in a patient affected by lymphedema after breast cancer treatment, showed altered functions, including lymphatic reflux, reduced pumping activity, tortuous and leaky vasculature, and incomplete ICG uptake [207–208].

Another potential application for ICG is the evaluation of sentinel lymph node (SLN) for nodal staging of cancer, especially for breast and melanoma cancer. In general, the identification of SLN is carried out with ^99m^Tc, but the use of both mg-doses and micro-doses of ICG allowed to estimate the SLN at 5 cm of depth during breast surgery, with good concordance with scintigraphic imaging [207,209–210]. The identification of SLN is being evaluated also in other tumors, such as prostate, gastric, lung, and skin cancers [211–214].

The third application, which has been recently tested, is the use of ICG to characterize primary breast cancer lesions: in fact, the clearance of ICG from tissues can be influenced by the presence of leaked vessels that is a common feature of tumors, and could help in the discrimination of malignant lesion from benign disease. Human breast imaging has been performed by using tomographic techniques, that are based on the measurements of NIR absorption and fluorescence before and after administration of ICG and on a mathematical model of light propagation and fluorescence generation in tissues, in order to generate a 3D map of optical properties that delineates and characterizes breast lesions.

There are very few works about this application: in two pilot studies a clear absorption increase was observed in the malignant tumors due to accumulation of injected-ICG [215–216] and in another study [217], late contrast of ICG, compared to early contrast, was able to better differentiate malignant and benign lesions, with good agreement with conventional mammography or ultrasonography [207].

Summarizing, ICG fluorescence for lymphatic vasculature diagnosis and assessment of its function is safe and minimally invasive, and can be performed repeatedly. Moreover, reported examples highlighted the possibility to use a micro-dose of ICG dye to perform imaging, reaching the micro-dose of radiolabeled probe, that is generally defined as “less than 1/100th of the dose calculated to yield a pharmacological effect of the test substance to a maximum dose of < 100 micrograms” [218]. In addition, the acquisition is very fast, lasting milliseconds, and ICG allows to visualize even the contractility of lymph vessels, a finding difficult to detect with the other techniques.

ICG has been also used in some clinical trials dealing with other cancer types. For example, it has been tested to evaluate the best perfused area for the surgeon to perform transection and anastomosis during colorectal surgery (ClinicalTrials.gov Identifier: NCT02598414): this is the principal cause of morbidity and mortality in this kind of surgery. The imaging has been performed by intravenous injection of 2.5 mg/ml of ICG, and this procedure has been repeated twice during surgery, before and after the anastomosis. The microvascularization at the anastomosis site has been assessed using a robotic fluorescence imaging device (FireFly™). In addition, ICG has also been proposed for the evaluation of the extent of kidney tumors, with the aim to scheme the surgery (ClinicalTrials.gov Identifier: NCT01281488). The primary objective has been the determination of the optimal dose of ICG for the visualization of renal carcinoma, and the secondary objectives were focused on report of peri-operative outcomes, such as resected margins, perfusion and renal function, operation time and correlation with intraoperative ultrasound imaging and preoperative imaging.

As well as ICG, other fluorescent dyes or tracers are used in clinical trials, even if they are still ongoing and no results are yet available; some examples are reported below.

In a glioma clinical trial (ClinicalTrials.gov Identifier: NCT02473380), fluorescence spectroscopy has been used to visualize glioma mass during surgery by using 5 amino-levulinic hydrochloride (GLIOLAN) as a-specific contrast agent. It is an un-fluorescent oral preparation that is absorbed by cells, especially by glioma cells or cancer cells, where it is principally converted in Protoporfirin IX (PPIX). When excited by a blue light, PPIX emits red fluorescence, while healthy tissues remain blue. This differential diagnosis allows surgeons to perform a more accurate resection of cancer lesions, without affecting normal brain portions.

Beside the use of a-specific contrast agents, some clinical trials entail the use of targeted fluorescent agents, with the aim to visualize a specific molecule expressed by tumor cells. An example of these clinical trials is the use of a near infra-red labeled folate analog, the OTL38, in order to detect renal cancer carcinoma at the resection margins in partial nephrectomy and lymph node or other sites of metastasis in radical surgery (ClinicalTrials.gov Identifier: NCT02645409) to better and faster evaluate the outcome of operation.

The last example is the use of Bevacizumab, which has been labeled with IRDye800CW and has been used during endoscopy in patients with hereditary colon cancer syndromes like Familial Adenomatous Polyposis (ClinicalTrials.gov Identifier: NCT02113202), that showed overexpression of VEGF, the target molecules of the monoclonal antibody Bevacizumab. The study is based on the idea that bevacizumab-IRDye800CW accumulates in VEGF expressing adenomas after its administration, enabling tumor visualization using a newly developed near-infrared (NIR) fluorescence endoscopy platform (NL43407.042.13).

The same tracer has also been used in another trial for the visualization of rectal cancer, in order to detect the presence of the drug target, in this case VEGF, to identify those patients who could benefit from a specific targeted treatment (ClinicalTrials.gov Identifier: NCT01972373).

All these examples seem to support the importance of fluorescence imaging during surgery or endoscopic imaging, with the aim to obtain fast and safe information about cancer mass and margins, its characterization and to facilitate complete surgical removal.

## LABELING OF NEWLY DISCOVERED CELLULAR TARGETS

Different processes and molecules can be easily visualized by using fluorescent probes, but in the future the need will arise to detect newly discovered cellular targets. An example of this process has been described in a very recent article of Salanti A. et al [219]. In this work, the authors have explored the anchor protein VAR2CSA, involved in the binding of Malaria-infected erythrocytes to placental syncytioblasts, in order to avoid the splenic clearance of infected cells. VAR2CSA binds a specific type of glycosaminoglycan (GAG), the chondroitin sulfate GAG, called CSA, that was found to be expressed in 95% of tumors generated by hematopoietic, epithelial and mesenchymal cells. The authors labeled the recombinant VAR2CSA protein with two different fluorophores, the Alexa488 and Alexa750 to perform *in vitro* and *in vivo* imaging, respectively. Data showed rapid *in vitro* internalization within 30 minutes in a colon cancer cell line (Colo205), and specific *in vivo* uptake, after intravenous administration, in xenograft mice bearing prostate (PC3 cell line) or melanoma (B16 cell line) cancer lesions. These data supported the possibility to use new targets in addition to the already used ones to visualize tumors. Moreover, since VAR2CSA is rapidly internalized after binding with CSA, this mechanism could be explored as a targeted strategy for the specific drug delivery to tumor.

In this paragraph we will explore the different possibilities among newly discovered cellular targets; moreover, an elucidation of the possibilities to conjugate fluorophores and targets and the criteria for the right choice of labeling molecules will be provided.

Among the newly discovered potential targets there are many different molecules ranging from polysaccharides, proteins, amino acids (where substantially there are no currently available fluorescent probes), peptides, as well as larger particles, such as nanoparticles and vesicles. Moreover, inasmuch as miRNAs have lately acquired a great importance in cancer development, establishment and progression, they could include new targets to study for the development of new probes for cancer detection and visualization.

A fluorescent molecule, to be considered a suitable label for *in vivo* imaging, needs to satisfy different requirements:

- it should be excited without contemporaneous excitation of surrounding tissues, and the emission should be detectable with conventional devices;
- it should have high brightness, molar absorption (calculated as absorbance divided by the absorption in the path through tissues) and quantum yield (calculated as number of emitted photons occurring per number of absorbed photons);
- it should have chemical groups capable of binding different kinds of molecules;
- it should be soluble in different aqueous fluids, stable in the most common conditions and non-toxic.

Biomolecules can be labelled by fluorescence, as shown in the examples reported in the paragraphs above, using organic molecules, semiconductor nanocrystals, and fluorescent proteins.

The field of organic fluorescent molecules is bigger, and is characterized by availability of dyes with different wavelengths, ranging between near-ultraviolet to near infra-red. However, despite the broad range of dyes, only a small part of the spectrum can be used to perform *in vivo* imaging. In fact, in the body tissues are present molecules that are natural chromophores, which strongly affect the incident or emitted flux of exogenous fluorescent probes by absorbing photons. This phenomenon decreases by increasing the wavelength of radiation, and is dependent upon the tissue features and its physical state [220]. For examples, tissues that contain high level of hemoglobin strongly absorb photons in the range of blue-green emission (e.g. 400-470 nm), but this phenomenon becomes negligible for wavelengths higher than ~600 nm. In fact, once passed this threshold, the tissues become quite more transparent to photons, and red and near-infra red emitting fluorescent dyes result the best choice for *in vivo* imaging FLI strategies.

Among fluorescent molecules emitting at >500 nm, that is the minimum wavelength to avoid absorption by hemoglobin, Rhodamines, AlexaFluor and Cyanine dyes are the most commonly used fluorophores for molecule labeling.

Rhodamine [221] is the oldest synthetic class of dyes used to label molecules. They generally are characterized by high molar absorptivity in the visible region and high brightness. Modern rhodamines are linked either non-covalently or covalently to target molecules, and the most exploited fluorophores are Rhodamine G [222] and Rhodamine 800 [223]. The former has an excitation peak at 526 nm, and an emission at 548 nm, whereas the latter is characterized by a double peak of excitation at 623 and 682, a double peak of emission at 700 and 774 nm, good photostability, a very low anisotropy and an acceptable fluorescence quantum yield.

AlexaFluor [224] is a family of dyes owned by Molecular Probe, Inc. They are synthesized by sulfonation of already existing molecules, such as coumarin, rhodamine, fluorescein and cyanine dyes, making them negatively charged and hydrophilic. In general, the Alexa-Fluor derivative dyes are characterized by higher stability, more brightness and less sensitivity to pH compared to the organic dyes with comparable fluorescent spectra. Their emission spectra range between 442 and 805 nm, with a great availability of probes in the red and near-infra red window. However, their use is limited due to the costs.

Cyanines [221] are fluorescent compounds having two aromatic or heterocyclic rings linked by a polymethine chain with conjugated carbon-carbon double bonds. Currently they are the main source of labeling fluorophores, showing an excitation and emission bands ranging between 600 and 900 nm. Moreover, they show linear and easy synthesis, possibility to tune their wavelengths, high near infra-red absorption and emission, and large molar absorptivity. They show some disadvantages as well: short fluorescence lifetimes, low fluorescent quantum yields and sometimes they undergo aggregation in aqueous solutions, determining low final fluorescence intensities. Some of the drawbacks could be sort out. For example, by using macromolecules or organic solvents, the solubility could be improved, or the introduction of alkyl sulfonate groups could allow to ameliorate water solubility, quantum yield and stability. Moreover, it is possible to insert additional functional groups to facilitate their covalent binding to target. Examples of compounds belonging to this fluorescent class are Cy5 and ICG. Cy5 is excited at 650 nm, and emits at 670 nm. Thanks to its fluorescent spectra in near-infra red region and its high extinction coefficient it has become particularly attractive for probe labeling. In addition, its derivative Cy5.5 shows a more shifted spectrum in NIR, being excited at 678 nm and emitting at 694 nm. ICG [225] is a tricarbocyanine dye with a molecular weight of 751.4 Da, able to emit at 800 nm and longer, depending on its chemical environment and physical condition. Even if it tends to aggregate and shows a very low fluorescence efficiency in aqueous solution, the use of solvent and the binding to proteins or molecules reduce these problems, and its approval by FDA for clinical use [207,226], makes it a useful tool during intraoperative decision making (as already explained in the previous paragraph).

Fluorophores able to emit at <500 nm could be used in *in vitro* studies, as well as in restricted *in vivo* applications where photon attenuation due to absorption by the tissues is not a limiting factor, e.g. endoscopic procedures [227].

The second possibility to label molecules is using semiconductor nanocrystals [228], such as Quantum Dots (QD). They are small light-emitting particles on the nanometer scale, formed by a core of CdSe, generally overcoated with ZnS, but also with CdS, ZnSe, CdTe and PbSe. Their optical proprieties are determined by the materials used for the coating and the emission color is dependent upon their size. For example, a small nanocrystal of 2-nm core emits green fluorescence -maximum 550 nm, 15% quantum yield- whereas a larger one, of about 4-nm core, emits red fluorescence -maximum 630 nm, 6% quantum yield-, reaching until 2 um of wavelength in the far-red. Moreover, they are characterized by high quantum yield and molar extinction coefficients (10-100 fold higher than organic dyes), broad spanning of emission, high resistance to photo-bleaching and to photo- and chemical-degradation [229]. In addition, a great advantage of QDs is that their surface could be functionalized with the aim to target biological molecules, either through electrostatic and hydrogen-bonding interactions or through a specific ligand-receptor interaction. The cytotoxicity of QDs is comparable with those of more commonly used fluorophores [230], and QDs have other advantages compared to organic dyes [231]: they are characterized by narrow emission band, the excitation and emission spectral position are tunable in relation to the size (quantum size effect), the molar absorption is higher, the quantum yield is good for both visible and near-infra red wavelengths (in contrast, organic dyes have fluorescence quantum yields that are high in the visible light range but are at best moderate in the NIR wavelength range) and the lifespan of fluorescence is longer (that last feature can sometimes represent a disadvantage, because it could make harder to quantify the signal in relation to time). In addition, QDs allow to perform two-photon imaging and multiplexing.

Even considering all these advantages, could these agents definitively replace fluorescent dyes? The answer is no, even if they can become complementary, especially in the field of *in vivo* imaging. Fluorescent dyes are an easy, safe and quite inexpensive strategy to label probes, they are commercially available, there are well-established protocols for their use and their proprieties are largely studied. Conversely, QD use is limited because their commercial availability is restricted, sometimes the signal quantification is difficult and there is the need of approved protocols for their functionalization.

The last option for the labeling of molecules is the use of recombinant fluorescent proteins [232]. These proteins represent a useful tool to visualize processes or molecules *in vivo*; in fact they can be designed to respond to a much greater variety of biological events and signals. However, they are generally used through their introduction into cell genome, and not as molecule labels, and no examples are reported for their use as labels in oncological imaging applications. Conversely, Luciferases, used to perform Bioluminescence Imaging, were sometimes utilized to label probes (such as in the examples reported for CEA antigens in paragraph 3.1). This strategy is not the best choice, because enzymes are bigger than other dyes and, in addition, Luciferase requires the administration of a substrate to be metabolized to generate light.

Once identified the right fluorescent dye, and performed the appropriate chemical preparation, the labeled probe needs to be tested for specificity, in order to establish if the molecule modifications have affected the behavior of the probe, and for sensitivity, to evaluate if the fluorophore provides a sufficiently high signal to noise ratio.

Although the number of available fluorescent dyes is high, there are still limitations, both in the physico-chemical characteristics and in fluorophore wavelength availability, especially in the near infra-red region, that is the best light windows to perform *in vivo* imaging. Thus, there is a strong need for new dyes with improved water solubility, higher quantum yield, wavelength emission >600÷700 nm and possibility to link them to target molecules.

## BOX 1. PROBES FOR VESSELS VISUALIZATION

An alternative to integrin for targeting angiogenesis consists in probes allowing the visualization of whole vascular network.

A “smart probe” called AngioSense has been developed (Perkin Elmer). It is a NIR fluorescent macromolecule capable of visualizing the vasculature and the blood vessel density [233–235], remaining localized over time in the vasculature and permitting to indirectly assess blood vessel density, vascular leakage and angiogenesis, allowing the study of treatment effects on vasculature as well. Moreover, two different fluorophores with different wavelengths (680 and 750 nm) are available to label this probe, making it possible to simultaneously follow two different probes, thus studying two different processes, such as angiogenesis and proliferation or hypoxia, in the same animal.

In the same way, AngioSPARK, a new family of highly fluorescent near infrared nano-particles specifically designed for *in vivo* imaging are already commercially available (Perkin Elmer). AngioSPARKs contain an iron oxide core coated with pegylated fluorescent molecules to specifically produce functionalized biocompatible nanoparticles that remain localized in the vasculature for a quite long period of time, allowing to perform imaging of blood vessels and angiogenesis and of vascular leakage at the site of inflammation and in cancer by optical imaging and by MRI.

In addition, a newly proposed approach entails the use of the tomato protein (*L. esculentum*) lectin, to obtain a new probe named TLectinSense680TM, useful for vascular labeling. This protein has high binding affinity for glycoprotein N-acetylglucosamines on the surface of vascular endothelial cells, allowing the targeting of vasculature and enabling blood vessels and angiogenesis imaging by further conjugation with a near infra-red emitting fluorophore. In a recent work, it has been used for improving the efficacy, early detection and monitoring of the effect of anti-angiogenic therapies [237].

Lastly, LI-COR company has developed a new probe (called IRDye 800CW PEG Contrast Agent) for Enhanced Permeability Retention (EPR) effect, that is of particular importance in cancer: the endothelium of vessels present in the tumor microenvironment is often discontinuous, and molecules are able to diffuse into the surrounding tumor tissue. In addition to EPR effect, the lymphatic drainage is reduced. As a consequence, larger molecules tend to accumulate. IRDye 800CW PEG Contrast Agent is a non-specific imaging agent for the visualization of EPR in tumor, for example in oral cancer [238].

## CONCLUSIONS

To date, clinical trials have a crucial role in determining whether fluorescent imaging probes could be effective and safe, but human *in vivo* studies are limited, being mainly based on the use of nuclear imaging and magnetic resonance techniques. In this scenario murine models can help in developing new fluorescent probes and in understanding their potential.

In the meantime, different kinds of probes have already been developed for optical imaging of tumor metabolism, proliferation, hypoxia, angiogenesis, invasiveness, inflammation and apoptosis, as well as for the early identification of tumor establishment and its characterization in relation to the response to treatments. Pre-clinical studies demonstrated the versatility of optical imaging strategies in the non-invasive assessment of these specific biomarkers, emphasizing the importance of their early detection to set up more powerful therapeutic strategies.

Clinical translation of these procedures has two main limitations: the first involves the use of fluorescence imaging in humans and the physical limitations that could be overcome by introducing new fluorophores or endoscopic or intra-operatory imaging procedures; on the other hand several of the probes described in this review have not yet been approved for human studies although significant steps have been made to study their sensitivity and specificity and the absence of toxic or adverse effects. Once these agents will be approved for clinical practice, optical imaging will have the potential to improve complete surgical resection of tumors by real-time image-guided surgery, as well as, to *in vivo* characterize the tumor without the need of potentially harmful procedures using ionizing radiations.

Fluorescent probes can also be efficiently used in animal models of human cancers for the validation of new targets and new targeted imaging strategies. The use of probes already approved for clinical applications in nuclear or radiological imaging could help in increasing the translational potential of the pre-clinical results regarding tumor characterization and stratification for the improvement of response to treatment. At the same time the identification of fluorophores applicable in the clinical setting and the development of new devices for fluorescence imaging in humans could speed up the clinical translation of these new probes.

## References

[1] Cerenkov P (1937). Visible Radiation Produced by Electrons Moving in a Medium with Velocities Exceeding that of Light. Physical Review.

[2] Contag CH, Bachmann MH (2002). Advances in *In vivo* Bioluminescence Imaging of Gene Expression. Annual Review of Biomedical Engineering.

[3] Ntziachristos V (2006). Fluorescence molecular imaging. Annual Review of Biomedical Engineering.

[4] Day RN, Davidson MW (2009). The fluorescent protein palette: tools for cellular imaging. Chemical Society Reviews.

[5] Contag CH, Ross BD (2002). It's not just about anatomy: *In vivo* bioluminescence imaging as an eyepiece into biology. Journal of Magnetic Resonance Imaging.

[6] Merényi G, Lind J, Eriksen TE (1990). Luminol chemiluminescence: Chemistry, excitation, emitter. Journal of Bioluminescence and Chemiluminescence.

[7] Tanha K, Pashazadeh AM, Pogue BW (2015). Review of biomedical Cerenkov luminescence imaging applications. Biomedical Optics Express.

[8] Spinelli AE, Boschi F (2015). Novel biomedical applications of Cerenkov radiation and radioluminescence imaging. Physica Medica.

[9] Spinelli AE, Ferdeghini M, Cavedon C, Zivelonghi E, Calandrino R, Fenzi A, Sbarbati A, Boschi F (2013). First human Cerenkography. Journal of Biomedical Optics.

[10] Levin CS (2008). New Imaging Technologies to enhance the molecular sensitivity of Positron Emission Tomography. Proceeding of the IEEE.

[11] Lecchi M, Ottobrini L, Martelli C, Del Sole A, Lucignani G (2007). Instrumentation and probes for molecular and cellular imaging. The Quarterly Journal of Nuclear Medicine and Molecular Imaging.

[12] Plathow C, Weber WA (2008). Tumor Cell Metabolism Imaging. Journal of Nuclear Medicine.

[13] Dang CV (2012). Links between metabolism and cancer. Genes & Development.

[14] Lloyd PG, Hardin CD, Sturek M (1999). Examining glucose transport in single vascular smooth muscle cells with a fluorescent glucose analog. Physiological Research.

[15] Zhang M, Zhang Z, Blessington D, Li H, Busch TM, Madrak V, Miles J, Chance B, Glickson JD, Zheng G (2003). Pyropheophorbide 2-deoxyglucosamide: a new photosensitizer targeting glucose transporters. Bioconjugate Chemistry.

[16] Cheng Z, Levi J, Xiong Z, Gheysens O, Keren S, Chen X, Gambhir SS (2006). Near-infrared fluorescent deoxyglucose analogue for tumor optical imaging in cell culture and living mice. Bioconjugate Chemistry.

[17] Kovar JL, Volcheck W, Sevick-Muraca E, Simpson MA, Olive DM (2009). Characterization and performance of a near-infrared 2-deoxyglucose optical imaging agent for mouse cancer models. Analytical Biochemistry.

[18] Guo J, Dua C, Shana L, Zhua H, Xuea B, Qianc Z, Achilefu S, Gu Y (2012). Comparison of near-infrared fluorescent deoxyglucose probes with different dyes for tumor diagnosis *in vivo*. Contrast Media & Molecular Imaging.

[19] Tseng JC, Wang Y, Banerjee P, Kung AL (2012). Incongruity of Imaging Using Fluorescent 2-DG Conjugates Compared to 18F-FDG in Preclinical Cancer Models. Molecular Imaging and Biology.

[20] Evan GI, Vousden KH (2011). Proliferation, cell cycle and apoptosis in cancer. Nature.

[21] Soloviev D, Lewis D, Honess D, Aboagye E (2012). [18F]FLT: an imaging biomarker of tumour proliferation for assessment of tumor response to treatment. European Journal of Cancer.

[22] Woolf DK, Berensford M, Li SP, Dowsett M, Sanghera B, Wong WL, Sonoda L, Detre S, Amin V, Ah-See ML, Miles D, Makris A (2014). Evaluation of FLT-PET-CT as an imaging biomarker of proliferation in primary breast cancer. British Journal of Cancer.

[23] Shields AF, Grierson JR, Dohmen BM, Machulla HJ, Stayanoff JC, Lawhorn-Crews JM, Obradovich JE, Muzik O, Mangner TJ (1998). Imaging proliferation *in vivo* with [F-18]FLT and positron emission tomography. Nature Medicine.

[24] Shields AF (2003). PET Imaging with 18F-FLT and Thymidine Analogs: Promise and Pitfalls. Journal of Nuclear Medicine.

[25] McKinley ET, Ayers GD, Smith RA, Saleh SA, Zhao P, Washington MK, Coffey RJ, Manning HC (2013). Limits of [18F]-FLT PET as a Biomarker of Proliferation in Oncology. PLoS One.

[26] Peterson JD Development of a Near Infrared Fluorescent Labeled Agent for Assessing Bombesin Receptor Expression in Cancer. Application Note Perkin Elmer.

[27] Orton SP, Morris JD, Smith BA, Leevy M (2012). *In vivo* Fluorescence Imaging of Tumor Proliferation Using Pre-labeled Cancer Cells and a Targeted Probe. Carestream Molecular Imaging.

[28] Ke CY, Mathias CJ, Green MA (2003). The folate receptor as a molecular target for tumor-selective radionuclide delivery. Nuclear Medicine and Biology.

[29] Zhao X, Li H, Lee RJ (2008). Targeted drug delivery *via* folate receptors. Expert Opinion in Drug Delivery.

[30] Low PS, Henne WA, Doorneweerd DD (2008). Discovery and development of folic-acid-based receptor targeting for imaging and therapy of cancer and inflammatory diseases. Accounts of Chemical Research.

[31] Yano K, Nimura H, Mitsumori N, Takahashi N, Kashiwagi H, Yanaga K (2012). The efficiency of micrometastasis by sentinel node navigation surgery using indocyanine green and infrared ray laparoscopy system for gastric cancer. Gastric Cancer.

[32] Hirche C, Engel H, Hirche Z, Doniga S, Herold T, Kneser U, Lehnhardt M, Hünerbein M (2014). Real-Time Lymphography by Indocyanine Green Fluorescence: Improved Navigation for Regional Lymph Node Staging. Annals of Plastic Surgery.

[33] Moon WK, Lin Y, O'Loughlin T, Tang Y, Kim DE, Weissleder R, Tung CH (2003). Enhanced tumor detection using a folate receptor-targeted near-infrared fluorochrome conjugate. Bioconjugate Chemistry.

[34] Liu F, Deng D, Chen X, Qian Z, Achilefu S, Gu Y (2010). Folate-polyethylene glycol conjugated near-infrared fluorescence probe with high targeting affinity and sensitivity for *in vivo* early tumor diagnosis. Molecular Imaging and Biology.

[35] Kirchherr AK, Briel A, Mader K (2009). Stabilization of indocyanine green by encapsulation within micellar systems. Molecular Pharmaceutics.

[36] Zheng C, Zheng M, Gong P, Jia D, Zhang P, Shi B, Sheng Z, Ma Y, Cai L (2012). Indocyanine green-loaded biodegradable tumor targeting nanoprobes for *in vitro* and *in vivo* imaging. Biomaterials.

[37] Chen J, Corbin IR, Li H, Cao W, Glickson JD, Zheng G (2007). Ligand conjugated low-density lipoprotein nanoparticles for enhanced optical cancer imaging *in vivo*. Journal of American Chemical Society.

[38] Schroeder JE, Shweky I, Shmeeda H, Banin U, Gabizon A (2007). Folate-mediated tumor cell uptake of quantum dots entrapped in lipid nanoparticles. Journal of Control Release.

[39] Jie Pan, Si-Shen Feng (2009). Targeting and imaging cancer cells by Folate-decorated, quantum dots (QDs)- loaded nanoparticles of biodegradable polymers. Biomaterials.

[40] Yang Y, An F, Liu Z, Zhang X, Zhou M, Li W, Hao X, Lee CS, Zhang X (2012). Ultrabright and ultrastable nearinfrared dye nanoparticles for *in vitro* and *in vivo* bioimaging. Biomaterials.

[41] Melillo G (2006). Inhibiting Hypoxia-Inducible Factor 1 for Cancer Therapy. Molecular Cancer Research.

[42] Semenza G (2002). Signal transduction to hypoxia-inducible factor 1. Biochemical Pharmacology.

[43] Ivan M, Kondo K, Yang H, Kim W, Valiando J, Ohh M, Salic A, Asara JM, Lane WS, Kaelin WG (2001). HIFalpha targeted for VHL-mediated destruction by proline hydroxylation: implications for O2 sensing. Science.

[44] Höckel M, Vaupel P (2001). Biological consequences of tumor hypoxia. Seminars in Oncology.

[45] Lo Dico A, Valtorta S, Martelli C, Belloli S, Gianelli U, Tosi D, Bosari S, Degrassi A, Russo M, Raccagni I, Lucignani G, Moresco RM, Ottobrini L (2014). Validation of an Engineered Cell Model for *In vitro* and *In vivo* HIF-1 Evaluation by Different Imaging Modalities. Molecular Imaging and Biology.

[46] Lo Dico A, Martelli C, Valtorta S, Raccagni I, Diceglie C, Belloli S, Gianelli U, Vaira V, Politi LS, Bosari S, Lucignani G, Moresco RM, Ottobrini L (2015). Identification of imaging biomarkers for the assessment of tumour response to different treatments in a preclinical glioma model. European Journal of Nuclear Medicine and Molecular Imaging.

[47] Kaluz S, Kaluzová M, Liao SY, Lerman M, Stanbridge EJ (2009). Transcriptional control of the tumor- and hypoxia-marker carbonic anhydrase 9: a one transcription factor (HIF-1) show?. Biochimica et Biophysica Acta.

[48] Koo YE, Cao Y, Kopelman R, Koo SM, Brasuel M, Philbert MA (2004). Real-time measurements of dissolved oxygen inside live cells by organically modified silicate fluorescent nanosensors. Analytical Chemistry.

[49] Sorg BS, Moeller BJ, Donovan O, Cao Y, Dewhirst MW (2005). Hyperspectral imaging of hemoglobin saturation in tumor microvasculature and tumor hypoxia development. Journal of Biomedical Optics.

[50] Cai H, Conti PS (2013). RGD-based PET tracers for imaging receptor integrin v3 expression. Journal of Labeled Compound & Radiopharmaceutical.

[51] Rapisarda A, Melillo G (2012). Role of the VEGF/VEGFR Axis in Cancer Biology and Therapy. Advances in Cancer Research.

[52] Backer MV, Patel V, Jehning BT, Backer JM (2006). Self-assembled “dock and lock” system for linking payloads to targeting proteins. Bioconjugate Chemistry.

[53] Chen K, Li ZB, Wang H, Cai W, Chen X (2008). Dual-modality optical and positron emission tomography imaging of vascular endothelial growth factor receptor on tumor vasculature using quantum dots. European Journal of Nuclear Medicine and Molecular Imaging.

[54] Terwisscha van Scheltinga AG, van Dam GM, Nagengast WB, Ntziachristos V, Hollema H, Herek JL, Schröder CP, Kosterink JG, Lub-de Hoog MN, de Vries EG (2011). Intraoperative near-infrared fluorescence tumor imaging with vascular endothelial growth factor and human epidermal growth factor receptor 2 targeting antibodies. Journal of Nuclear Medicine.

[55] Weis SM, Cheresh DA (2011). Integrins in Angiogenesis and Cancer. Cold Spring Harbor Perspectives in Medicine.

[56] Achilefu S, Bloch S, Markiewicz MA, Zhong T, Ye Y, Dorshow RB, Chance B, Liang K (2005). Synergistic effects of light-emitting probes and peptides for targeting and monitoring integrin expression. Proceedings of the National Academy of Sciences (PNAS).

[57] Chen X, Conti PS, Moats RA (2004). *In vivo* near-infrared fluorescence imaging of integrin alphavbeta3 in brain tumor xenografts. Cancer Research.

[58] Huang R, Vider J, Kovar JL, Olive DM, Mellinghoff IK, Mayer-Kuckuk P, Kircher MF, Blasberg RG (2012). Integrin v3-Targeted IRDye 800CW Near-Infrared Imaging of Glioblastoma. Clinical Cancer Research.

[59] Wu Y, Cai W, Chen X (2006). Near-infrared fluorescence imaging of tumor integrin alpha v beta 3 expression with Cy7-labeled RGD multimers. Molecular Imaging and Biology.

[60] Jin ZH, Josserand V, Razkin J, Garanger E, Boturyn D, Favrot MC, Dumy P, Coll JL (2006). Noninvasive optical imaging of ovarian metastases using Cy5-labeled RAFT-c(-RGDfK-)4. Molecular Imaging.

[61] Jin ZH, Razkin J, Josserand V, Boturyn D, Grichine A, Texier I, Favrot MC, Dumy P, Coll JL (2007). *In vivo* noninvasive optical imaging of receptor-mediated RGD internalization using self-quenched Cy5-labeled RAFT-c(-RGDfK-)(4). Molecular Imaging.

[62] Boturyn D, Coll JL, Garanger E, Favrot MC, Dumy P (2004). Template assembled cyclopeptides as multimeric system for integrin targeting and endocytosis. Journal of American Chemical Society.

[63] Wu Y, Zhang X, Xiong Z, Cheng Z, Fisher DR, Liu S, Gambhir SS, Chen X (2005). microPET imaging of glioma integrin v3 expression using (64)Cu-labeled tetrameric RGD peptide. Journal of Nuclear Medicine.

[64] Montet X, Montet-Abou K, Reynolds F, Weissleder R, Josephson L (2006). Nanoparticle imaging of integrins on tumor cells. Neoplasia.

[65] Schellenberger EA, Sosnovik D, Weissleder R, Josephson L (2004). Magneto/optical annexin V, a multimodal protein. Bioconjugate Chemistry.

[66] Kitov PI, Bundle DR (2003). On the Nature of the Multivalency Effect: A Thermodynamic Model. Journal of American Chemical Society.

[67] Hardman R (2006). A toxicologic review of quantum dots: toxicity depends on physicochemical and environmental factors. Environmental Health Perspectives.

[68] Lia C, Wang W, Wu Q, Ke S, Houston J, Sevick-Muraca E, Dong L, Chow D, Charnsangavej C, Gelovani JG (2006). Dual optical and nuclear imaging in human melanoma xenografts using a single targeted imaging probe. Nuclear Medicine and Biology.

[69] Kimura RH, Miao Z, Cheng Z, Gambhir SS, Cochran JR (2010). A Dual-Labeled Knottin Peptide for PET and Near-Infrared Fluorescence Imaging of Integrin Expression in Living Subjects. Bioconjugate Chemistry.

[70] Pallaghy PK, Nielsen KJ, Craik DJ, Norton RS (1994). A common structural motif incorporating a cystine knot and a triple-stranded beta-sheet in toxic and inhibitory polypeptides. Protein Science.

[71] Kossodo S, Pickarski M, Lin SA, Gleason A, Gaspar R, Buono C, Ho G, Blusztajn A, Cuneo G, Zhang J, Jensen J, Hargreaves R, Coleman P (2010). Dual *in vivo* quantification of integrin-targeted and protease-activated agents in cancer using fluorescence molecular tomography (FMT). Molecular Imaging and Biology.

[72] Huang CW, Li Z, Conti PS (2011). *In vivo* Near-Infrared Fluorescence Imaging of Integrin alpha2beta1 in Prostate Cancer with Cell-Penetrating-Peptide-Conjugated DGEA Probe. Journal of Nuclear Medicine.

[73] Aina OH, Marik J, Gandour-Edwards R, Lam KS (2005). Near-infrared optical imaging of ovarian cancer xenografts with novel alpha 3-integrin binding peptide “OA02”. Molecular Imaging.

[74] Yao N, Xiao W, Wang X, Marik J, Park SH, Takada Y, Lam KS, Kurth MJ (2009). Discovery of targeting ligands for breast cancer cells using the one-bead one-compound combinatorial method. Journal of Medical Chemistry.

[75] Carpenter RD, Andrei M, Aina OH, Lau EY, Lightstone FC, Liu R, Lam KS, Kurth MJ (2009). Selectively targeting T- and B-cell lymphomas: a benzothiazole antagonist of alpha4beta1 integrin. J Med Chem.

[76] Peng L, Liu R, Marik J, Wang X, Takada Y, Lam KS (2006). Combinatorial chemistry identifies high-affinity peptidomimetics against alpha4beta1 integrin for *in vivo* tumor imaging. Nature Chemical Biology.

[77] Duffy MJ (1992). The role of proteolytic enzymes in cancer invasion and metastasis. Clinical & Experimental Metastasis.

[78] Tan GJ, Peng ZK, Lu JP, Tang FQ (2013). Cathepsins mediate tumor metastasis. World Journal of Biological Chemistry.

[79] Palermo C, Joyce JA (2008). Cysteine cathepsin proteases as pharmacological targets in cancer. Trends in Pharmacological Sciences.

[80] Paulick MG, Bogyo M (2008). Application of activity-based probes to the study of enzymes involved in cancer progression. Current Opinion in Genetics & Development.

[81] Edgington LE, Bogyo M (2013). *In vivo* imaging and biochemical characterization of protease function using fluorescent activity-based probes. Current Protocols in Chemical Biology.

[82] Lee S, Park K, Kim K, Choi K, Kwon IC (2008). Activatable imaging probes with amplified fluorescent signals. Chemical Communication.

[83] Blum G, von Degenfeld G, Merchant MJ, Blau HM, Bogyo M (2007). Noninvasive optical imaging of cysteine protease activity using fluorescently quenched activity-based probes. Nature Chemical Biology.

[84] Weissleder R, Tung CH, Mahmood U, Bogdanov A (1999). *In vivo* imaging of tumors with protease-activated near-infrared fluorescent probes. Nature Biotechnology.

[85] Jaffer FA, Kim DE, Quinti L, Tung CH, Aikawa E, Pande AN, Kohler RH, Kohler RH, Shi GP, Libby P, Weissleder R (2007). Optical visualization of cathepsin K activity in atherosclerosis with a novel, proteaseactivatable fluorescence sensor. Circulation.

[86] Williams RM, Flesken-Nikitin A, Ellenson LH, Connolly DC, Hamilton TC, Nikitin AY, Zipfel WR (2010). Strategies for High-Resolution Imaging of Epithelial Ovarian Cancer by Laparoscopic Nonlinear Microscopy. Translational Oncology.

[87] Keereweer S, Mol IM, Vahrmeijer AL, Van Driel PBBA, Baatenburg de Jong RJ, Kerrebijn JDF, Löwik CW (2012). Dual wavelength tumor targeting for detection of hypopharyngeal cancer using near-infrared optical imaging in an animal model. International Journal of Cancer.

[88] Hensley HH, Roder NA, O'Brien SW, Bickel LE, Xiao F, Litwin S, Connolly DC (2012). Combined *In vivo* Molecular and Anatomic Imaging for Detection of Ovarian Carcinoma-Associated Protease Activity and Integrin Expression in Mice. Neoplasia.

[89] Abd-Elgaliel WR, Cruz-Monserrate Z, Logsdonbc CD, Ching-Hsuan Tung (2011). Molecular imaging of Cathepsin E-positive tumors in mice using a novel protease-activatable fluorescent probe. Molecular BioSystem.

[90] Shuman Moss LA, Jensen-Taubman S, Stetler-Stevenson WG (2012). Matrix metalloproteinases: changing roles in tumor progression and metastasis. American Journal of Pathology.

[91] Tsukuba T, Okamoto K, Yasuda Y, Morikawa W, Nakanishi H, Yamamoto K (2000). New functional aspects of cathepsin D and cathepsin E. Molecules and Cells.

[92] Veiseh M, Gabikian P, Bahrami SB, Veiseh O, Zhang M, Hackman RC, Ravanpay AC, Stroud MR, Kusuma Y, Hansen SJ, Kwok D, Munoz NM, Sze RW (2007). Tumor Paint: A Chlorotoxin:Cy5. 5 Bioconjugate for Intraoperative Visualization of Cancer Foci. Cancer Research.

[93] Deshane J, Garner CC, Sontheimer H (2003). Chlorotoxin inhibits glioma cell invasion *via* matrix metalloproteinase-2. Journal of Biological Chemistry.

[94] Lyons SA, O'Neal J, Sontheimer H (2002). Chlorotoxin, a scorpion-derived peptide, specifically binds to gliomas and tumors of neuroectodermal origin. Glia.

[95] Hockaday DC, Shen S, Fiveash J, Raubitschek A, Colcher D, Liu A, Alvarez V, Mamelak AN (2005). Imaging glioma extent with 131I-TM-601. Journal of Nuclear Medicine.

[96] Deshane J, Garner CC, Sontheimer H (2003). Chlorotoxinb inhibits glioma cell invasion *via* matrix metalloproteinase-2. Journal of Biological Chemistry.

[97] McFerrin MB, Sontheimer H (2006). A role for ion channels in glioma cell invasion. Neuron Glia Biology.

[98] Bremer C, Bredow S, Mahmood U, Weissleder R, Tung CH (2001). Optical imaging of matrix metalloproteinase-2 activity in tumors: feasibility study in a mouse model. Radiology.

[99] Chuang CH, Chuang KH, Wang HE, Roffler SR, Shiea JT, Tzou SC, Cheng TC, Kao CH, Wu SY, Tseng WL, Cheng CM, Hou MF, Wang JM (2011). *In vivo* Positron Emission Tomography Imaging of Protease Activity by Generation of a Hydrophobic Product from a Noninhibitory Protease Substrate. Clinical Cancer Research.

[100] Zheng G, Chen J, Stefflova K, Jarvi M, Li H, Wilson BC (2007). Photodynamic molecular beacon as an activatable photosensitizer based on protease-controlled singlet oxygen quenching and activation. Proceedings of the National Academy of Sciences (PNAS).

[101] Yhee JY, Kim SA, Koo H, Son S, Ryu JH, Youn IC, Choi K, Kwon IC, Kim K (2012). Optical imaging of cancer-related proteases using near-infrared fluorescence matrix metalloproteinase-sensitive and cathepsin B-sensitive probes. Theranostics.

[102] Lee MS, Kim YH, Kim YJ, Kwon SH, Bang JK, Lee SM, Song YS, Hahm DH, Shim I, Han D, Her S (2011). Pharmacokinetics and biodistribution of human serum albumin-TIMP-2 fusion protein using near-infrared optical imaging. J of Pharmacy & Pharmaceutical Sciences.

[103] Waschkau B, Faust A, Schäfers M, Bremer C (2013). Performance of a new fluorescence-labeled MMP inhibitor to image tumor MMP activity *in vivo* in comparison to an MMP-activatable probe. Contrast Media & Molecular Imaging.

[104] Coussens LM, Werb Z (2002). Inflammation and cancer. Nature.

[105] Mantovani A, Allavena P, Sica A, Balkwill F (2008). Cancer-related inflammation. Nature.

[106] Foersch S, Neufert C, Neurath MF, Waldner MJ (2013). Endomicroscopic Imaging of COX-2 Activity in Murine Sporadic and Colitis-Associated Colorectal Cancer. Diagnostic and Therapeutic Endoscopy.

[107] Soslow RA, Dannenberg AJ, Rush D, Woerner BM, Khan KN, Masferrer J, Koki AT (2000). COX-2 Is Expressed in Human Pulmonary, Colonic, and Mammary Tumors. Cancer.

[108] Wang D, DuBois RN (2010). The role of COX-2 in intestinal inflammation and colorectal cancer. Oncogene.

[109] Rizzo MT (2011). Cyclooxygenase-2 in oncogenesis. Clinica Chimica Acta.

[110] Zhang H, Fan J, Wang J, Dou B, Zhou F, Cao J, Qu J, Cao Z, Zhao W, Peng X (2013). Fluorescence discrimination of cancer from inflammation by molecular response to COX-2 enzymes. Journal of American Chemical Society.

[111] Hirai M, Minematsu H, Kondo N, Oie K, Igarashi K, Yamazaki N (2007). Accumulation of liposome with Sialyl Lewis X to inflammation and tumor region: application to *in vivo* bio-imaging. Biochemical and Biophysical Research Communication.

[112] Yoo JS, Lee SC, Jow ZY, Koh PY, Chang YT (2014). A macrophage-specific fluorescent probe for intraoperative lymph node staging. Cancer Research.

[113] Gounaris E, Tung CH, Restaino C, Maehr R, Kohler R, Joyce JA, Ploegh HL, Barrett TA, Weissleder R, Khazaie K (2008). Live Imaging of Cysteine-Cathepsin Activity Reveals Dynamics of Focal Inflammation, Angiogenesis, and Polyp Growth. PloS ONE.

[114] Balducci A, Wen Y, Zhang Y, Helfer BM, Hitchens TK, Meng WS, Wesa AK, Janjic JM (2013). A novel probe for the non-invasive detection of tumor-associated inflammation. OncoImmunology.

[115] Kadayakkara DK, Ranganathan S, Young W-B, Ahrens ET (2012). Assaying macrophage activity in a murine model of inflammatory bowel disease using fluorine-19 MRI. Laboratory Investigation.

[116] Hitchens TK, Ye Q, Eytan DF, Janjic JM, Ahrens ET, Ho C (2011). 19F MRI detection of acute allograft rejection with *in vivo* perfluorocarbon labeling of immune cells. Magnetic Resonance Medicine.

[117] Tseng JC, Kung AL (2012). *In vivo* Imaging of Inflammatory Phagocytes. Chemistry & Biology.

[118] Li Y, Zhu H, Kuppusamy P, Roubaud V, Zweier JL, Trush MA (1998). Validation of Lucigenin (Bis-N-methylacridinium) as a Chemilumigenic Probe for Detecting Superoxide Anion Radical Production by Enzymatic and Cellular Systems. Journal of Biology and Chemisrty.

[119] Kerr JFR, Winterford CM, Harmon BV (1994). Apoptosis. Its significance in cancer and cancer Therapy. Cancer.

[120] Johnstone RW, Ruefli AA, Lowe SW (2002). Apoptosis: a link between cancer genetics and chemotherapy. Cell.

[121] Reutelingsperger CP, Hornstra G, Hemker HC (1985). Isolation and partial purification of a novel anticoagulant from arteries of human umbilical cord. European Journal of Biochemistry.

[122] Koopman G, Reutelingsperger CP, Kuijten GAM, Keehnen RM, Pals ST, van Oers MH (1994). Annexin V for flow cytometric detection of phosphatidylserine expression on B cells undergoing apoptosis. Blood.

[123] Agarwal A, Mackey MA, El-Sayed MA, Bellamkonda RV (2011). Remote triggered release of doxorubicin in tumors by synergistic application of thermosensitive liposomes and gold nanorods. ACS Nano.

[124] Petrovsky A, Schellenberger E, Josephson L, Weissleder R, Bogdanov A (2003). Near-infrared fluorescent imaging of tumor apoptosis. Cancer Research.

[125] Manning HC, Merchant NB, Foutch AC, Virostko JM, Wyatt SK, Shah C, McKinley ET, Xie J, Mutic NJ, Washington MK, LaFleur B, Tantawy MN, Peterson TE (2008). Molecular imaging of therapeutic response to epidermal growth factor receptor blockade in colorectal cancer. Clinical Cancer Research.

[126] van Tilborg GA, Mulder WJ, Chin PT, Storm G, Reutelingsperger CP, Nicolay K, Strijkers GJ (2006). Annexin A5-conjugated quantum dots with a paramagnetic lipidic coating for the multimodal detection of apoptotic cells. Bioconjugate Chemistry.

[127] Corsten MF, Hofstra L, Narula J, Reutelingsperger CPM (2006). Counting Heads in the War against Cancer: Defining the Role of Annexin A5 Imaging in Cancer Treatment and Surveillance. Cancer Research.

[128] Olsson M, Zhivotovsky B (2011). Caspases and cancer. Cell Death and Differentiation.

[129] Bullok K, Piwnica-Worms D (2005). Synthesis and characterization of a small, membrane-permeant, caspase activatable far-red fluorescent peptide for imaging apoptosis. Journal of Medical Chemistry.

[130] Bullok KE, Maxwell D, Kesarwala AH, Gammon S, Prior JL, Snow M, Stanley S, Piwnica-Worms D (2007). Biochemical and *in vivo* characterization of a small, membrane-permeant, caspase-activatable farred fluorescent peptide for imaging apoptosis. Biochemistry.

[131] Edgington LE, Berger AB, Blum G, Albrow VE, Paulick MG, Lineberry N, Bogyo M (2009). Noninvasive optical imaging of apoptosis by caspase-targeted activity-based probes. Nature Medicine.

[132] Stefflova K, Chen J, Marotta D, Li H, Zheng G (2006). Photodynamic therapy agent with a built-in apoptosis sensor for evaluating its own therapeutic outcome in situ. Journal of Medicinal Chemistry.

[133] MacDonald IJ, Morgan J, Bellnier DA, Paszkiewicz GM, Whitaker JE, Litchfield DJ, Dougherty TJ (1999). Subcellular localization patterns and their relationship to photodynamic activity of pyropheophorbide-a derivatives. Photochemistry and Photobiology.

[134] Chen J, Stefflova K, Niedre MJ, Wilson BC, Chance B, Glickson JD, Zheng G (2004). Protease-triggered photosensitizing beacon based on singlet oxygen quenching and activation. Journal of American Chemical Society.

[135] Stefflova K, Chen J, Li H, Zheng G (2006). Targeted photodynamic therapy agent with a built-in apoptosis sensor for *in vivo* near-infrared imaging of tumor apoptosis triggered by its photosensitization in situ. Molecular Imaging.

[136] Hammarstrom S (1999). The carcinoembryonic antigen (CEA) family: structures, suggested functions and expression in normal and malignant tissues. Seminars In Cancer Biology.

[137] Cuesta AM, Sanchez-Martin D, Sanz L, Bonet J, Compte M, Kremer L, Blanco FJ, Oliva B, Alvarez-Vallina L (2009). *In vivo* tumor targeting and imaging with engineered trivalent antibody fragments containing collagen-derived sequences. PLoS One.

[138] Kaushal S, McElroy MK, Luiken GA, Talamini MA, Moossa AR, Hoffman RM, Bouvet M (2008). Fluorophore-conjugated anti-CEA Antibody for the Intraoperative Imaging of Pancreatic and Colorectal Cancer. Journal of Gastrointestinal Surgery.

[139] Lisy MR, Goermar A, Thomas C, Pauli J, Resch-Genger U, Kaiser WA, Hilger I (2008). *In vivo* Near-infrared Fluorescence Imaging of Carcinoembryonic Antigen-expressing Tumor Cells in Mice. Radiology.

[140] Han S, Mu Y, Zhu Q, Gao Y, Li Z, Jin Q, Jin W (2012). Au:CdHgTe quantum dots for *in vivo* tumor-targeted multispectral fluorescence imaging. Analytical and Bioanalytical Chemistry.

[141] Venisnik KM, Olafsen T, Loening AM, Iyer M, Gambhir SS, Wu AM (2006). Bifunctional antibody-Renilla luciferase fusion protein for *in vivo* optical detection of tumors. Protein Engineering, Design & Selection.

[142] Venisnik KM, Olafsen T, Gambhir SS, Wu AM (2007). Fusion of Gaussia luciferase to an engineered anti-carcinoembryonic antigen (CEA) antibody for *in vivo* optical imaging. Molecular Imaging and Biology.

[143] Chen LD, Liu J, Yu XF, He M, Pei XF, Tang ZY, Wang QQ, Pang DW, Li Y (2008). The biocompatibility of quantum dot probes used for the targeted imaging of hepatocellular carcinoma metastasis. Biomaterials.

[144] Williams SA, Singh P, Isaacs JT, Denmeade SR (2007). Does PSA play a role as a promoting agent during the initiation and/or progression of prostate cancer?. Prostate.

[145] Williams SA, Jelinek CA, Litvinov I, Cotter RJ, Isaacs JT, Denmeade SR (2011). Enzymatically active prostate-specific antigen promotes growth of human prostate cancers. Prostate.

[146] Ho G, Morin J, Delaney J, Cuneo G, Yared W, Rajopadhye M, Peterson JD, Kossodo S (2013). Detection and quantification of enzymatically active prostate-specific antigen *in vivo*. Journal of Biomedical Optics.

[147] Nakajima T, Mitsunaga M, Bander NH, Heston WD, Choyke PL, Kobayashi H (2011). Targeted, activatable, *in vivo* fluorescence imaging of prostate-specific membrane antigen (PSMA) positive tumors using the quenched humanized J591 antibody-indocyanine green (ICG) conjugate. Bioconjugate Chemistry.

[148] Gao X, Cui Y, Levenson RM, Chung LW, Nie S (2004). *in vivo* cancer targeting and imaging with semiconductor quantum dots. Nature Biotechnology.

[149] Shi C, Zhu Y, Xie Z, Qian W, Hsieh CL, Nie S, Su Y, Zhau HE, Chung LW (2009). Visualizing human prostate cancer cells in mouse skeleton using bioconjugated near-infrared fluorescent quantum dots. Urology.

[150] Chen Y, Pullambhatla M, Banerjee SR, Byun Y, Stathis M, Rojas C, Slusher BS, Mease RC, Pomper MG (2012). Synthesis and biological evaluation of low molecular weight fluorescent imaging agents for the prostate-specific membrane antigen. Bioconjugate Chemistry.

[151] Carpenter G, Cohen S (1990). Epidermal growth factor. Journal of Biological Chemistry.

[152] Yarden Y (2001). The EGFR family and its ligands in human cancer. Signalling mechanisms and therapeutic opportunities. European Journal of Cancer.

[153] Spano JP, Lagorce C, Atlan D, Milano G, Domont J, Benamouzig R, Attar A, Benichou J, Martin A, Morere JF, Raphael M, Penault-Llorca F, Breau JL (2005). Impact of EGFR expression on colorectal cancer patient prognosis and survival. Annals of Oncology.

[154] Soukos NS, Hamblin MR, Keel S, Fabian RL, Deutsch TF, Hasan T (2001). Epidermal growth factor receptor-targeted immunophotodiagnosis and photoimmunotherapy of oral precancer *in vivo*. Cancer Research.

[155] Rosenthal EL, Kulbersh BD, King T, Chaudhuri TR, Zinn KR (2007). Use of fluorescent labeled antiepidermal growth factor receptor antibody to image head and neck squamous cell carcinoma xenografts. Molecular and Cancer Therapy.

[156] Wang K, Wang K, Li W, Huang T, Li R, Wang D (2009). Characterizing Breast Cancer Xenograft Epidermal Growth Factor Receptor Expression by Using Near-Infrared Optical Imaging. Acta Radiologica.

[157] Hama Y, Urano Y, Koyama Y, Choyke PL, Kobayashi H (2007). Activatable fluorescent molecular imaging of peritoneal metastases following pretargeting with a biotinylated monoclonal antibody. Cancer Research.

[158] Wang W, Wang EQ, Balthasar JP (2008). Monoclonal Antibody Pharmacokinetics and Pharmacodynamics. Nature.

[159] Friedman M, Nordberg E, Hoiden-Guthenberg I, Brismar H, Adams GP, Nilsson FY, Carlsson J, Ståhl S (2007). Phage display selection of Affibody molecules with specific binding to the extracellular domain of the epidermal growth factor receptor. Protein Engineering, Design & Selection.

[160] Miao Z, Ren G, Liu H, Jiang L, Cheng Z (2010). Cy5.5-labeled Affibody molecule for near-infrared fluorescent optical imaging of epidermal growth factor receptor positive tumors. Journal of Biomedical Optics.

[161] Gong H, Kovar J, Little G, Chen H, Olive DM (2010). *in vivo* imaging of xenograft tumors using an epidermal growth factor receptor-specific affibody molecule labeled with a near-infrared fluorophore. Neoplasia.

[162] Qi S1, Miao Z, Liu H, Xu Y, Feng Y, Cheng Z (2012). Evaluation of Four Affibody-Based Near-Infrared Fluorescent Probes for Optical Imaging of Epidermal Growth Factor Receptor Positive Tumors. Bioconjugate Chemistry.

[163] Horak E, Heitner T, Robinson MK, Simmons HH, Garrison J, Russeva M, Furmanova P, Lou J, Zhou Y, Yuan QA, Weiner LM, Adams GP, Marks JD (2005). Isolation of scFvs to *in vitro* produced extracellular domains of EGFR family members. Cancer Biotherapy & Radiopharmaceutical.

[164] Zhou Y, Drummond DC, Zou H, Hayes ME, Adams GP, Kirpotin DB, Marks JD (2007). Impact of single chain Fv antibody fragment affinity on nanoparticle targeting of epidermal growth factor receptor expressing tumor cells. Journal of Molecular Biology.

[165] Yang L, Mao H, Wang YA, Cao Z, Peng X, Wang X, Duan H, Ni C, Yuan Q, Adams G, Smith MQ, Wood WC, Gao X (2008). Single Chain Epidermal Growth Factor Receptor Antibody Conjugated Nanoparticles for *in vivo* Tumor Targeting and Imaging. Small.

[166] Ke S, Wen X, Gurfinkel M, Charnsangavej C, Wallace S, Sevick-Muraca EM, Li C (2003). Near-infrared optical imaging of epidermal growth factor receptor in breast cancer xenografts. Cancer Research.

[167] Kovar JL, Johnson MA, Volcheck WM, Chen J, Simpson MA (2006). Hyaluronidase expression induces prostate tumor metastasis in an orthotopic mouse model. Americal Journal of Pathology.

[168] Adams KE, Ke S, Kwon S, Liang F, Fan Z, Lu Y, Hirschi K, Mawad ME, Barry MA, Sevick-Muraca EM (2007). Comparison of visible and near-infrared wavelength-excitable fluorescent dyes for molecular imaging of cancer. Journal of Biomedical Optics.

[169] Perez EA, Cortés J, Gonzalez-Angulo AM, Bartlett JM (2014). HER2 testing: current status and future directions. Cancer Treatment Review.

[170] Owens MA, Horten BC, Da Silva MM (2004). HER2 amplification ratios by fluorescence in situ hybridization and correlation with immunohistochemistry in a cohort of 6556 breast cancer tissues. Clinical Breast Cancer.

[171] Hilger I, Leistner Y, Berndt A, Fritsche C, Haas KM, Kosmehl H, Kaiser WA (2004). Near-infrared fluorescence imaging of HER-2 protein over-expression in tumour cells. European Radiology.

[172] Lee SB, Hassan M, Fisher R, Chertov O, Chernomordik V, Kramer-Marek G, Gandjbakhche A, Capala J (2008). Affibody Molecules for *in vivo* Characterization of HER2-Positive Tumors by Near-Infrared Imaging. Clinical Cancer Research.

[173] Gee MS, Upadhyay R, Bergquist H, Alencar H, Reynolds F, Maricevich M, Weissleder R, Josephson L, Mahmood U (2008). Human Breast Cancer Tumor Models: Molecular Imaging of Drug Susceptibility and Dosing during HER2/neu-targeted Therapy. Radiology.

[174] Ogawa M, Regino CA, Choyke PL, Kobayashi H (2009). *in vivo* target-specific activatable near-infrared optical labeling of humanized monoclonal antibodies. Molecular Cancer Therapeutics.

[175] Ogawa M, Kosaka N, Choyke PL, Kobayashi H (2009). Tumor-specific detection of an optically targeted antibody combined with a quencher-conjugated neutravidin “quencher-chaser”: a dual “quench and chase” strategy to improve target to nontarget ratios for molecular imaging of cancer. Bioconjugate Chemistry.

[176] Ogawa M, Kosaka N, Longmire MR, Urano Y, Choyke PL, Kobayashi H (2009). Fluorophore quencher based activatable targeted optical probes for detecting *in vivo* cancer metastases. Molecular Pharmacology.

[177] Koyama Y, Hama Y, Urano Y, Nguyen DM, Choyke PL, Kobayashi H (2007). Spectral fluorescence molecular imaging of lung metastases targeting HER2/neu. Clinical Cancer Research.

[178] Ogawa M, Kosaka N, Choyke PL, Kobayashi H (2009). *in vivo* Molecular Imaging of Cancer with a Quenching Near-Infrared Fluorescent Probe Using Conjugates of Monoclonal Antibodies and Indocyanine Green. Cancer Research.

[179] Tada H, Higuchi H, Wanatabe TM, Ohuchi N (2007). *in vivo* real-time tracking of single quantum dots conjugated with monoclonal anti-HER2 antibody in tumors of mice. Cancer Research.

[180] Narayanan N, Cuneo G, Morin J, Vasquez K, Rajopadhye M, Yared W, Peterson JD, Kossodo SC (2012). Development of a red fluorescent labeled agent for assessing HER2 expression *in vitro* and *in vivo*. Cancer Res.

[181] Wikman M, Steffen AC, Gunneriusson E, Tolmachev V, Adams GP, Carlsson J, Ståhl S (2004). Selection and characterization of HER2/neu-binding affibody ligands. Protein Engineering, Design & Selection.

[182] Zielinski R, Hassan M, Lyakhov I, Needle D, Chernomordik V, Garcia-Glaessner A, Ardeshirpour Y, Capala J, Gandjbakhche A (2012). Affibody-DyLight Conjugates for *In vivo* Assessment of HER2 Expression by Near-Infrared Optical Imaging. PLoS ONE.

[183] Gao J, Chen K, Miao Z, Ren G, Chen X, Gambhir SS, Cheng Z (2011). Affibody-based nanoprobes for HER2-expressing cell and tumor imaging. Biomaterials.

[184] Lee SB, Hassan M, Fisher R, Chertov O, Chernomordik V, Kramer-Marek G, Gandjbakhche A, Capala J (2008). Affibody molecules for *in vivo* characterization of HER2-positive tumors by near-infrared imaging. Clinical Cancer Research.

[185] Gong H, Kovar J, Little G, Chen H, Olive DM (2010). *In vivo* imaging of xenograft tumors using an epidermal growth factor receptor-specific affibody molecule labeled with a near-infrared fluorophore. Neoplasia.

[186] Weng KC, Noble CO, Papahadjopoulos-Sternberg B, Chen FF, Drummond DC, Kirpotin DB, Wang D, Hom YK, Hann B, Park JW (2008). Targeted tumor cell internalization and imaging of multifunctional quantum dot-conjugated immunoliposomes *in vitro* and *in vivo*. Nano Letters.

[187] Greish K (2010). Enhanced permeability and retention (EPR) effect for anticancer nanomedicine drug targeting. Methods in Molecular Biology.

[188] Maeda H (2012). Macromolecular therapeutics in cancer treatment: the EPR effect and beyond. Journal of Control Release.

[189] Zhang X, Yee D (2002). Insulin-like Growth Factor Binding Protein-1 (IGFBP-1) Inhibits Breast Cancer Cell Motility. Cancer Research.

[190] Zhang H, Yee D (2004). The therapeutic potential of agents targeting the type I insulin-like growth factor receptor. Expert Opinion on Investigational Drugs.

[191] Sachdev D, Zhang X, Matise I, Gaillard-Kelly M, Yee D (2010). The type I insulin-like growth factor receptor regulates cancer metastasis independently of primary tumor growth by promoting invasion and survival. Oncogene.

[192] Burtrum D, Zhu Z, Lu D, Anderson DM, Prewett M, Pereira DS, Bassi R, Abdullah R, Hooper AT, Koo H, Jimenez X, Johnson D, Apblett R (2003). A fully human monoclonal antibody to the insulin-like growth factor I receptor blocks ligand-dependent signaling and inhibits human tumor growth *In vivo*. Cancer Research.

[193] Goetsch L, Gonzalez A, Leger O, Beck A, Pauwels PJ, Haeuw JF, Corvaia N (2005). A recombinant humanized anti-insulin-like growth factor receptor type I antibody (h7C10) enhances the antitumor activity of vinorelbine and anti-epidermal growth factor receptor therapy against human cancer xenografts. International Journal of Cancer.

[194] Zhang H, Zeng X, Li Q, Gaillard-Kelly M, Wagner CR, Yee D (2009). Fluorescent tumour imaging of type I IGF receptor in vivo: comparison of antibody-conjugated quantum dots and small-molecule fluorophore. British Journal of Cancer.

[195] Sun LC, Coy DH (2011). Somatostatin Receptor-Targeted Anti-Cancer Therapy. Current Drug Delivery.

[196] Beiderwellen KJ, Poeppel TD, Hartung-Knemeyer V, Buchbender C, Kuehl H, Bockisch A, Lauenstein TC (2013). Simultaneous 68Ga-DOTATOC PET/MRI in Patients With Gastroenteropancreatic Neuroendocrine Tumors: Initial Results. Investive Radiology.

[197] Becker A, Hessenius C, Licha K, Ebert B, Sukowski U, Semmler W, Semmler W, Wiedenmann B, Grötzinger C (2001). Receptor-targeted optical imaging of tumors with near-infrared fluorescent ligands. Nature Biotechnology.

[198] Fottner C, Mettler E, Goetz M, Schirrmacher E, Anlauf M, Strand D, Schirrmacher R, Klöppel G, Delaney P, Schreckenberger M, Galle PR, Neurath MF, Kiesslich R (2010). *In vivo* molecular imaging of somatostatin receptors in pancreatic islet cells and neuroendocrine tumors by miniaturized confocal laser-scanning fluorescence microscopy. Endocrinology.

[199] Dorsam RT, Gutkind JS (2007). G-protein-coupled receptors and cancer. Nature Reviews Cancer.

[200] Lappano R, Maggiolini M (2011). G protein-coupled receptors: novel targets for drug discovery in cancer. Nature Reviews Drug Discovery.

[201] Ferrand A, Wang TC (2006). Gastrin and cancer: A review. Cancer Letters.

[202] Ma L, Yu P, Veerendra B, Rold TL, Retzloff L, Prasanphanich A, Sieckman G, Hoffman TJ, Volkert WA, Smith CJ (2007). *In vitro* and *in vivo* evaluation of Alexa Fluor 680-bombesin[7–14]NH2 peptide conjugate, a high-affinity fluorescent probe with high selectivity for the gastrin-releasing peptide receptor. Molecular Imaging.

[203] Cai QY, Yu P, Besch-Williford C, Smith CJ, Sieckman GL, Hoffman TJ, MA L (2013). Near-infrared fluorescence imaging of gastrin releasing peptide receptor targeting in prostate cancer lymph node metastases. Prostate.

[204] Rosanò L, Spinella F, Bagnato A (2013). Endothelin 1 in cancer: biological implications and therapeutic opportunities. Nature Reviews Cancer.

[205] Holtke C, Waldeck J, Kopka K, Heindel W, Schober O, Schafers M, Bremer C (2009). Biodistribution of a nonpeptidic fluorescent endothelin A receptor imaging probe. Molecular Imaging.

[206] Büther K, Compeer MG, De Mey JG, Schober O, Schäfers M, Bremer C, Riemann B, Höltke C (2012). Assessment of endothelin-A receptor expression in subcutaneous and orthotopic thyroid carcinoma xenografts *in vivo* employing optical imaging methods. Endocrinology.

[207] Sevick-Muraca EM (2012). Translation of Near-Infrared Fluorescence Imaging Technologies: Emerging Clinical Applications. Annual Reviews in Medicine.

[208] Rasmussen JC, Tan IC, Marshall MV, Adams KE, Kwon S, Fife CE, Smith LA, Covington KR, Sevick-Muraca EM (2010). Human Lymphatic Architecture and Dynamic Transport Imaged Using Near-infrared Fluorescence. Translational Oncology.

[209] Sevick-Muraca EM, Sharma R, Rasmussen JC, Marshall MV, Wendt JA, Pham HQ, Bonefas E, Houston JP, Sampath L, Adams KE, Blanchard DK, Fisher RE, Chiang SB (2008). Imaging of lymph flow in breast cancer patients after microdose administration of a near-infrared fluorophore: feasibility study. Radiology.

[210] Kitai T, Inomoto T, Miwa M, Shikayama T (2005). Fluorescence navigation with indocyanine green for detecting sentinel lymph nodes in breast cancer. Breast Cancer.

[211] Polom K, Murawa D, Rho YS, Nowaczyk P, Hünerbein M, Murawa P (2011). Current trends and emerging future of indocyanine green usage in surgery and oncology: a literature review. Cancer.

[212] Zelken JA, Tufaro AP (2015). Current Trends and Emerging Future of Indocyanine Green Usage in Surgery and Oncology: An Update. Annals of Surgical Oncology.

[213] Schaafsma BE, Mieog JS, Hutteman M, van der Vorst JR, Kuppen PJ, Löwik CW, Frangioni JV, van de Velde CJ, Vahrmeijer AL (2011). The clinical use of indocyanine green as a near-infrared fluorescent contrast agent for image-guided oncologic surgery. Jornal of Surgical Oncology.

[214] Liberale G, Vankerckhove S, Caldon MG, Ahmed B, Moreau M, Nakadi IE, Larsimont D, Donckier V, Bourgeois P, Group R&D for the Clinical Application of Fluorescence Imaging of the Jules Bordets Institute (2016). Fluorescence Imaging After Indocyanine Green Injection for Detection of Peritoneal Metastases in Patients Undergoing Cytoreductive Surgery for Peritoneal Carcinomatosis From Colorectal Cancer: A Pilot Study. Annals of Surgery.

[215] Intes X, Ripoll J, Chen Y, Nioka S, Yodh AG, Chance B (2003). *In vivo* continuous-wave optical breast imaging enhanced with indocyanine green. Medical Physics.

[216] Corlu A, Choe R, Durduran T, Rosen MA, Schweiger M, Arridge SR, Schnall MD, Yodh AG (2007). Three-dimensional *in vivo* fluorescence diffuse optical tomography of breast cancer in humans. Optics Express.

[217] Poellinger A, Burock S, Grosenick D, Hagen A, Lüdemann L, Diekmann F, Engelken F, Macdonald R, Rinneberg H, Schlag PM (2011). Breast cancer: early- and late-fluorescence near-infrared imaging with indocyanine green--a preliminary study. Radiology.

[218] Usha Rani P, Naidu MUR (2008). Phase 0 - Microdosing strategy in clinical trials. Indian Journal of Pharmacology.

[219] Salanti A, Clausen TM, Agerbæk MØ, Al Nakouzi N, Dahlbäck M, Oo HZ, Lee S, Gustavsson T, Rich JR, Hedberg BJ, Mao Y, Barington L, Pereira MA (2015). Targeting Human Cancer by a Glycosaminoglycan Binding Malaria Protein. Cancer Cell.

[220] Jacques SL (2013). Optical properties of biological tissues: a review. Physical and Medical Biology.

[221] Goncalves MS (2009). Fluorescent labeling of Biomolecules with Organic Probes. Chemical Reviews.

[222] Asiria AM, Al-Amoudi MS, Al-Talhi TA, Al-Talhib AD (2011). Photodegradation of Rhodamine 6G and phenol red by nanosized TiO2 under solar irradiation. Journal of Saudi Chemical Society.

[223] Alessi A, Salvalaggio M, Ruzzon G (2013). Rhodamine 800 as reference substance for fluorescence quantum yield measurements in deep red emission range. Journal of Luminescence.

[224] Panchuk-Voloshina N, Haugland RP, Bishop-Stewart J, Bhalgat MK, Millard PJ, Mao F, Leung WY, Haugland RP (1999). Alexa dyes, a series of new fluorescent dyes that yield exceptionally bright, photostable conjugates. Journal of Histochemistry and Cytochemistry.

[225] Alander JT, Kaartinen I, Laakso A, Pätilä T, Spillmann T, Tuchin VV, Venermo M, Välisuo P (2012). A review of indocyanine green fluorescent imaging in surgery. International Journal of Biomedical Imaging.

[226] Boni L, David G, Mangano A, Dionigi G, Rausei S, Spampatti S, Cassinotti E, Fingerhut A (2015). Clinical applications of indocyanine green (ICG) enhanced fluorescence in laparoscopic surgery. Surgical Endoscopy.

[227] Hoetker MS, Goetz M (2013). Molecular imaging in endoscopy. United European Gastroenterol Journal.

[228] Bruchez M, Moronne M, Gin P, Weiss S, Alivisatos AP (1998). Semiconductor Nanocrystals as Fluorescent Biological Labels. Science.

[229] Medintz II, Uyeda HT, Goldman ER, Mattoussi H (2005). Quantum dot bioconjugates for imaging, labeling and sensing. Nature Materials.

[230] Bradburne CE, Delehanty JB, Boeneman Gemmill K, Mei BC, Mattoussi H, Susumu K, Blanco-Canosa JB, Dawson PE, Medintz IL (2013). Cytotoxicity of quantum dots used for *in vitro* cellular labeling: role of QD surface ligand, delivery modality, cell type, and direct comparison to organic fluorophores. Bioconjugate Chemistry.

[231] Resch-Genger U, Grabolle M, Cavaliere-Jaricot S, Nitschke R, Nann T (2008). Quantum dots *versus* organic dyes as fluorescent labels. Nature Methods.

[232] Shaner NC, Steinbach PA, Tsien RY (2005). A guide to choosing fluorescent proteins. Nature Methods.

[233] Ackermann M, Carvajal IM, Morse BA, Moreta M, O'Neil S, Kossodo S, Peterson JD, Delventhal V, Marsh HN, Furfine ES, Konerding MA (2011). Adnectin CT-322 inhibits tumor growth and affects microvascular architecture and function in Colo205 tumor xenografts. International Journal of Oncology.

[234] Zhang Q, Bindokas V, Shen J, Fan H, Hoffman RM, Xing HR (2011). Time-course imaging of therapeutic functional tumor vascular normalization by antiangiogenic agents. Mol Cancer Ther.

[235] Persigehl T, Ring J, Bremer C, Heindel W, Holtmeier R, Stypmann J, Claesener M, Hermann S, Schäfers M, Zerbst C, Schliemann C, Mesters RM, Berdel WE (2014). Non-invasive monitoring of tumor-vessel infarction by retargeted truncated tissue factor tTF-NGR using multi-modal imaging. Angiogenesis.

[236] Peterson JD Comparison of PerkinElmer Vascular Pre-clinical Fluorescent Imaging Agents in Oncology and Inflammation Research. Application Note Perkin Elmer.

[237] Morin J, Delaney J, Ho G, Cuneo G, Rajopadhye M, Yared W, Peterson JD, Kossodo SC (2012). Targeted *in vivo* imaging of tumor vasculature using a near infra-red labeled tomato lectin agent. WMIC (World Molecular Imaging Congress).

[238] Keereweer S, Mol IM, Kerrebijn JD, Van Driel PB, Xie B, Baatenburg de Jong RJ, Vahrmeijer AL, Löwik CW (2012). Targeting integrins and enhanced permeability and retention (EPR) effect for optical imaging of oral cancer. Journal of Surgical Oncology.

